# Microbial succession during the transition from active to inactive stages of deep-sea hydrothermal vent sulfide chimneys

**DOI:** 10.1186/s40168-020-00851-8

**Published:** 2020-06-30

**Authors:** Jialin Hou, Stefan M. Sievert, Yinzhao Wang, Jeffrey S. Seewald, Vengadesh Perumal Natarajan, Fengping Wang, Xiang Xiao

**Affiliations:** 1 State Key Laboratory of Microbial Metabolism, Joint International Research Laboratory of Metabolic & Developmental Sciences, School of Life Sciences and Biotechnology, Shanghai Jiao Tong University, Shanghai, China; 2 Biology Department, Woods Hole Oceanographic Institution, Woods Hole, MA USA; 3 Department of Marine Chemistry and Geochemistry, Woods Hole Oceanographic Institution, Woods Hole, MA USA; 4 School of Oceanography, Shanghai Jiao Tong University, Shanghai, China; 5 Southern Marine Science and Engineering Guangdong Laboratory (Zhuhai), Zhuhai, Guangdong China

**Keywords:** East Pacific Rise, Metagenome, Sulfide chimney, Microbial succession, *Nitrospirae*

## Abstract

**Background:**

Deep-sea hydrothermal vents are highly productive biodiversity hotspots in the deep ocean supported by chemosynthetic microorganisms. Prominent features of these systems are sulfide chimneys emanating high-temperature hydrothermal fluids. While several studies have investigated the microbial diversity in both active and inactive sulfide chimneys that have been extinct for up to thousands of years, little is known about chimneys that have ceased activity more recently, as well as the microbial succession occurring during the transition from active to inactive chimneys.

**Results:**

Genome-resolved metagenomics was applied to an active and a recently extinct (~ 7 years) sulfide chimney from the 9–10° N hydrothermal vent field on the East Pacific Rise. Full-length 16S rRNA gene and a total of 173 high-quality metagenome assembled genomes (MAGs) were retrieved for comparative analysis. In the active chimney (L-vent), sulfide- and/or hydrogen-oxidizing *Campylobacteria* and *Aquificae* with the potential for denitrification were identified as the dominant community members and primary producers, fixing carbon through the reductive tricarboxylic acid (rTCA) cycle. In contrast, the microbiome of the recently extinct chimney (M-vent) was largely composed of heterotrophs from various bacterial phyla, including *Delta*-/*Beta*-/*Alphaproteobacteria* and *Bacteroidetes*. *Gammaproteobacteria* were identified as the main primary producers, using the oxidation of metal sulfides and/or iron oxidation coupled to nitrate reduction to fix carbon through the Calvin-Benson-Bassham (CBB) cycle. Further analysis revealed a phylogenetically distinct *Nitrospirae* cluster that has the potential to oxidize sulfide minerals coupled to oxygen and/or nitrite reduction, as well as for sulfate reduction, and that might serve as an indicator for the early stages of chimneys after venting has ceased.

**Conclusions:**

This study sheds light on the composition, metabolic functions, and succession of microbial communities inhabiting deep-sea hydrothermal vent sulfide chimneys. Collectively, microbial succession during the life span of a chimney could be described to proceed from a “fluid-shaped” microbial community in newly formed and actively venting chimneys supported by the oxidation of reductants in the hydrothermal fluid to a “mineral-shaped” community supported by the oxidation of minerals after hydrothermal activity has ceased. Remarkably, the transition appears to occur within the first few years, after which the communities stay stable for thousands of years.

Video Abstract

## Introduction

Since the discovery of the first deep-sea hydrothermal vent (DSHV) near the Galapagos Islands in 1977 [1], more than 700 DSHV fields have been discovered and investigated along mid-oceanic ridges and other tectonically active areas of the ocean [2]. Water-rock reactions at high temperatures generated by subsurface magmatic heating transforms the seawater percolating into the ocean crust into hot, reduced, metal-rich hydrothermal fluids that vent from the seafloor. Therefore, DSHV systems are considered a critical conduit for the exchange of energy and matter between the Earth’s interior and the ocean [3, 4]. The hydrothermal vent field located on East Pacific Rise (EPR) at 9–10°N is an archetypical fast-spreading mid ocean ridge system (550 mm year^−1^) and as a result of a range of multi- and interdisciplinary studies over the last two and half decades represents one of the best studied hydrothermal systems [5]. More importantly, two volcanic eruptions have been documented at this location in 1991 and 2006 with dramatic effects on the geology, chemistry, and biology [5–9], providing unparalleled opportunities to study the evolution of a hydrothermal vent system with dynamic volcanic activity as well as the corresponding microbial succession.

Hydrothermal sulfide chimneys are typical vent structures, which are formed over short spatial and temporal scales by the precipitation of metal sulfides following the mixing of venting hydrothermal fluids with the surrounding cool, oxygenated seawater [10, 11]. The resulting thermodynamic and redox disequilibria provide conditions conducive for the growth of chemoautotrophic microorganisms, which colonize the interior and exterior parts of chimney walls according to their growth preference and contribute to the overall biomass production at DSHV [12, 13]. Previous microbiological investigations have shown that the microbial communities inhabiting active sulfide chimneys are diverse, including *Campylobacteria* (previously known as *Epsilonproteobacteria* [14]), *Aquificae*, *Gammaproteobacteria*, and some archaeal taxa [15–17]. Among them, *Campylobacteria* are frequently found as the dominant chemoautotrophic microorganisms of active hydrothermal vent chimneys [16, 18–25], often forming microbial mats covering the exterior of venting chimneys [21, 23, 26, 27]. Most *Campylobacteria* identified at DSHV are uncultured, but information on available isolates and incubation studies suggests that they are either thermophiles or mesophiles with the capability of chemoautotrophy driven by oxidation of H_2_ and/or H_2_S dissolved in the vent fluids [14, 28–32]. However, once hydrothermal activity ceases, the disappearance of the previously available energy sources and thermal gradients results in a pronounced shift of the microbial communities inhabiting the inactive chimneys to a community dominated by *Gamma*-, *Delta*-, *Alpha*-, *Betaproteobacteria*, and *Bacteroidetes* [19, 33–37]. Most of the studies on inactive chimneys have focused on revealing the microbial diversity and community structure using 16S rRNA-based analyses [19, 33–36]. Only recently, meta-“omic” approaches have been applied to study microbial metabolic potentials in extinct chimneys, which indicated that, among other findings, the oxidation of metal sulfides serves as important energy source mediated mainly by sulfur-oxidizing *Gammaproteobacteria* [37]. However, most of the inactive chimneys studied so far have been extinct for a long time (up to more than thousand years) [34–36]. Although the study by Meier et al [37] included a recently extinct chimney, dating put the age range of the chimney between 0 and160 years, and thus, the exact time when it became extinct is not known. Consequently, at present, less is known about the changes and succession in the microbial communities that occur in the immediate aftermath once an actively venting chimney becomes inactive.

Here, a sulfide sample from actively venting chimney (L-vent) and the other one from the inactive chimney (M-vent), which ceased venting 7 years before sampling as a result of the eruption in 2006 [5]), were collected in early 2014 from the hydrothermal vent field at 9–10° N EPR. The composition and the metabolic capabilities of their resident microbial communities were analyzed using genome-resolved metagenomics to elucidate the changes occurring during the transition from an active to an inactive chimney. Based on our data and available information from previous studies, we are proposing a conceptual model of microbial succession from “fluid-shaped” microbial community in newly formed and mature actively venting chimneys supported by the oxidation of reductants in the hydrothermal fluid to a “mineral-shaped” community supported by the oxidation of solid-phase minerals after hydrothermal activity has ceased.

## Material and methods

### Chimney and fluid samples collection

Sulfide chimney samples were collected by ROV Jason at the 9–10°N deep-sea hydrothermal vent field on the East Pacific Rise (EPR) during research cruise AT26-10 (December 2013 to January 2014). One sample (named L-vent) was collected from the flange of an active chimney at the L-vent structure (104.2789° W, 9.7712° N), venting hydrothermal fluid with temperature of 231 °C; the other one (named M-vent) was collected from a recently inactive chimney at the M-vent structure (104.2931° W, 9.8466° N) that became extinct as a result of the volcanic eruption in 2006 [5]. However, at the time of sampling in January 2014, the highly weathered M-vent chimney was found to emit warm fluid (35 °C) at a low flow rate. Chimney pieces were placed in sealed bioboxes to prevent mixing with ambient seawater during the recovery. Both samples were kept at − 70 °C immediately after sample retrieval on board and were then transported to the laboratory with dry ice and stored at − 80 °C before analysis. Three fluid samples were collected from L-vent and two from M-vent using isobaric gastight fluid samplers (IGT) to maintain fluids at seafloor pressure [38]. During sampling, the IGT snorkel with a thermocouple at its tip was positioned directly into the chimney orifices. When the temperature achieved a stable reading, the inlet valve of the sampler was opened for 2 min and then closed to maintain in situ pressure during recovery.

### Geochemistry measurements

Fluid samples were extracted from the retrieved IGT sampler for geochemical analysis either directly on board the ship (pH, CH_4_, H_2_, total dissolved sulfide) or upon return back to land in the shore-based laboratory (Mg^2+^, K^+^, Ca^2+^, Na^+^, Cl^−^). The pH was measured at 25 °C with an Ag/AgCl combination reference electrode. Dissolved CH_4_ and H_2_ concentrations were determined using a gas chromatograph equipped with a 5 Å molecular sieve packed column and serially connected thermal conductivity and flame ionization detectors following quantitative headspace extraction. Total dissolved sulfide (ΣH_2_S = H_2_S + HS^−^ + S^2−^) was determined potentiometrically using a sulfide-selective electrode. Dissolved Mg^2+^, K^+^, Ca^2+^, Na^+^, and Cl^−^ concentrations were analyzed by ion chromatography with suppressed conductivity detection. Estimates of overall analytical uncertainties (2 s) are ± 10% for H_2_, CH_4_, and ΣH_2_S, ± 3% for Na^+^, Mg^2+^, Ca^2+^, K^+^, Cl^−^, and SO^2−^_4_ concentrations, and ± 0.1 units for pH.

### DNA extraction, sequencing, assembly, and mapping

A modified SDS-based DNA extraction method [39] was used to recover sufficient high-quality DNA from the two chimney samples L-vent and M-vent. The paired-end sequencing was performed using a 2 × 100 bp Illumina HiSeq 2000 platform (TruSeq SBS KIT-HS V3, Illumina, at BGI-Shenzhen, China). Metagenome raw reads were trimmed with Sickle (v1.33) (https://github.com/najoshi/sickle) using the ‘-pe’ option with default parameters. Clean reads were merged and assembled using IDBA-UD (v1.1.3) with the following parameters: pre_correction, mink 52, maxk 92, step 8, and seed kmer 52 [40]. Clean reads were mapped onto their assembled contigs respectively using bowtie2 (v2.2.8) with --very sensitive mode [41]. The resulting sam file was sorted and converted to bam using samtools (v1.3.1), and depth of each contig was generated by using the *cytoscapeviz* script of multi-metagenome project [42].

### 16S rRNA gene reconstruction

Full-length 16S rRNA genes were reconstructed from clean reads of two metagenomes respectively by EMIRGE (v0.60.4) with default parameters and BLASTed against SILVA 123 SSURef_NR99 database with *e* values < 1 × 10^−10^ for taxonomic information [43, 44]. Relative abundance in phylum level (class level for *Proteobacteria*) is summarized based on the average sequencing depth of taxonomically assigned 16S rRNA genes from two samples, respectively.

### Annotation and statistic comparison of functional genes

For contigs larger than 1 Kb, open reading frames (ORFs) were predicted and translated by using prodigal (v2.6.3) with -p meta parameters [45], and the resulting amino acid sequences were uploaded to webserver GhostKOALA (KEGG Orthology And Links Annotation) in genus_prokaryotes + family_eukaryotes database with default parameters for KO annotation [46]. For cross checking, potential key genes involved in further analysis were also annotated in eggNOG database through emapper-eggnog (v0.0.1) as well as in Pfam 31.0, TIGRFAM 15.0, and custom databases via hmmsearch with cutoff *e* value < 1 × 10^−10^ [47–50]. Specific key genes and their accession in different databases are listed in Additional file 2: Table S7.

To quantitatively compare key genes between the two chimney samples, reads mapped to each gene were recruited by using featureCounts (v.1.5.0) [51], which was normalized by gene length, and the normalized relative abundance of key genes was determined for two samples:

$$ \frac{\mathrm{Mapped}\ \mathrm{reads}\ \mathrm{to}\ \mathrm{each}\ \mathrm{gene}}{\mathrm{Length}\ \mathrm{of}\ \mathrm{each}\ \mathrm{gene}}\times \left(\frac{1}{\sum \frac{\mathrm{Mapped}\ \mathrm{reads}\ \mathrm{to}\ \mathrm{each}\ \mathrm{gene}}{\mathrm{Length}\ \mathrm{of}\ \mathrm{each}\ \mathrm{gene}}}\right)\times {10}^6 $$

Statistical tests of key genes involved in carbon, nitrogen, and sulfur metabolism between the two metagenomes were performed by pairwise comparisons of their abundance by using two-sided Fisher’s exact test with confidence intervals at 95% significance using the Newcombe-Wilson method and Benjamini-Hochberg FDR multiple test correction in STAMP [52]. For those important catalytic genes, their taxonomy was assigned based on BLAST results in the NCBI NR database (updated in October 2018) with coverage > 50% and *e* value < 1 × 10^−10^. Taxonomic assignment was summarized at the phylum level (class for *Proteobacteria*). For each functional gene, taxonomic relative abundance is calculated based on the sum sequencing depth of genes with same taxonomic assignment in the total depth of this gene.

### Phylogenetic analysis of functional genes

Dissimilatory sulfite reductase (*dsr*) catalyzes either the reduction of sulfate in sulfate-reducing microorganisms or the reverse reaction in sulfide-oxidizing bacteria [53]. Here, the phylogeny of *dsrA* was used to distinguish between the two types [54]. The retrieved *dsrA* sequences from the current study were aligned with all high-quality *dsrA* amino acid sequences downloaded from NCBI NR database on July 2018 using MAFFT (v7.313) [55]. Gaps in the alignment were trimmed by the trimalAI (v1.4) with -automated1 and checked manually [56]. A phylogenic tree was generated by using IQ-tree (v1.6.6) using parameters: iqtree -m LG+C60+F+G -alrt 1000 -bb 1000 [57]. A similar approach was taken to phylogenetically characterize the marker gene *soxB* of the multienzyme sulfur-oxidizing (Sox) system and the *cyc2* gene.

### Metagenomic binning

The binning method used here is modified from Wang et al [58]. Contigs larger than 3 Kb from the two metagenomes were included to independently recover MAGs using MetaBAT2 (v2.12.1) and Maxbin (v2.2.1) with default parameters [59, 60]. The completeness and contamination of MAGs were estimated via CheckM (v1.0.9) with lineage-specific markers genes [61]. Because different independent automated binning methods reconstructed multiple similar MAGs from the same microbial taxa, we here used a modified method described in Park et al. to refine MAGs [62]. First, a pair of MAGs with identical taxonomic classification was combined into one single bin using the “merge” function of CheckM if integrated completeness and contamination would increase ≥ 10% and ≤ 1% respectively. Next, the contigs with divergent genomic properties (GC content, tetranucleotide and sequencing depth) and incongruent taxonomic classifications were filtered from their belonged MAGs by using the “outliers” method of RefineM (v0.0.22) [63]. Then, average amino acid identity (AAI) was calculated between each refined bin with CompareM (v0.0.23) (https://github.com/dparks1134/CompareM). We kept the one with higher completeness if two MAGs share AAI ≥ 99%. After filtering the reduplicate contigs in multiple MAGs, we kept qualified MAGs with completeness ≥ 70% and contamination ≤ 9% based on the evaluation from CheckM with parameter lineage-specific.

### Taxonomic assignment of MAGs

Qualified MAGs from two samples were phylogenetically assigned to appropriate taxonomic classifications based on a set of 37 concatenated universal single-copy protein sequences [63]. The reference genome dataset was downloaded from NCBI genome database in February 2018, including all available archaeal genomes and selected bacterial genomes with at least 10 from each order. Then, 37 marker genes were predicted in every reference genome and MAG by using reciprocal BLAST in the COG database, which was aligned separately by MAFFT (v7.313) with auto parameter and trimmed using trimalAI (v1.4) with automated1 [55, 56]. Phylogenetic tree was generated using RAxML (v8.2.8) with PROTGAMMALG model and 1000 bootstraps replicates [64]. The resulting phylogenetic tree was visualized using the Interactive Tree Of Life (iTOL) webtool [65]. In addition, further phylogenetic analyses were carried out for the MAGs assigned to *Nitrospirae*, *Gammaproteobacteria*, and *Campylobacteria*, respectively.

### Coverage, relative abundance, and replication rate of MAGs

The coverage of recovered MAGs in the communities were estimated through a method based on the unique marker gene *RpS3* [66], which were retrieved from these two chimney metagenomes by using hmmsearch (cutoff *e* value 1 × 10^−10^) in the Pfam 31.0 database [50]. Relative abundance for each MAG is determined by the proportion of length normalized depth of their binned contigs in all of contigs larger than 3 Kb. Index of replication (iRep) value is a quantitative measurement of the in situ replicate rates of bacterial MAGs based on their single-origin replication feature and sequencing coverage trend, which is a function of replicating population and number of replicate events [67]. Here, we determined the iRep value for those bacterial MAGs with ≤ 175 scaffolds per Mb by using the official script (https://github.com/christophertbrown/iRep).

### Metabolic analysis of MAGs

All retrieved MAGs were annotated by using eggnog-mapper-1.03 in the EggNOG database with *e* value < 1 × 10^−10^, which were further cross checked in the Pfam 31.0, TIGRfam 15.0, and custom hmmer databases with *e* value 10^−10^. Hydrogenases were predicted and classified based on hydrogenase classifier HydDB by using hmmsearch (*e* value cutoff < 1 × 10^−10^) [68]. After assignments of key genes, MAGs were assessed for the completeness of specific pathways and functions based on the canonical pathways available in KEGG Pathway Database (www.kegg.jp). The aerobic CO dehydrogenase (CODH) shares a high similarity with other enzymes from molybdenum hydroxylase family [69]. To prevent an overestimation of the potential for CO oxidation, we performed similar phylogenetic analysis for its catalyzed subunit *coxL* to confirm the presence of CODH in each MAG using previously compiled reference sequences from CO-oxidizing bacteria [69].

## Results

### Geochemical features of hydrothermal fluids

The measured temperature of the hydrothermal fluid discharged from the L-vent and M-vent chimney were 231 °C and 35 °C, respectively. Measured concentrations of dissolved chemical species in individual vent fluid samples are provided in Additional file 2: Table S1. For the L-vent, the composition of the three IGT fluid samples showed significant variability in the concentration of Mg, indicative of variable degrees of inadvertent seawater entrainment during sample collection. The composition of the endmember fluid at L-vent (Table 1) was calculated by plotting the concentration of a given chemical species against the measured Mg concentration in the same sample and extrapolating to zero-Mg, as is typically done for high-temperature submarine vent fluids [70]. Measured concentrations of Mg in the two fluids collected at M-vent showed little variation, consistent with entrainment of seawater in subseafloor environments prior to venting. Because we are interested in the chemical composition of fluids accessible to vent communities living within the chimney structures, the composition of M-vent fluids reported in Table 1 are not extrapolated to zero-Mg endmember values. At both vents, reported pH (25 °C) is the lowest measured value in the samples and is not extrapolated to zero-Mg.

**Table 1:** Concentrations of selected aqueous species in M-vent and L-vent fluids and seawater

	M-vent	L-vent	Seawater
	Measured	Endmember	
Temperature	35 °C	231 °C	2 °C
pH (25 °C)	4.8	5.2	7.8
K^+^ (mmol/kg)	10.7	18.9	9.95
Ca^2+^ (mmol/kg)	18.5	19.7	9.98
Mg^2+^ (mmol/kg)	39.2	0	53.0
Cl^−^ (mmol/kg)	549	528	537
SO_4_^2−^ (mmol/kg)	22.2	0	27.7
∑H_2_S (mmol/L)	0.00042	6.3	–
∑CO_2_ (mmol/kg)	21.6	11.5	2.3
CH_4_ (μmol/L)	24	83	–
H_2_ (mmol/L)	< 0.002	0.83	–

The L-vent concentrations are extrapolated endmember values (see text), while the M-vent values are measured concentrations

Endmember fluids venting through the L-vent structure were characterized by dissolved sulfide and H_2_ concentrations of 6.3 and 0.83 mM, respectively. In contrast, fluids exiting M-vent contained 0.00042 mM sulfide and H_2_ was below the detection limit of 2 μM. CH_4_ was also detected in both the L-vent and M-vent fluids at concentrations of 83 and 24 μM, respectively. Despite the stark difference in temperature, the measured pH (25 °C) showed similar values of 4.8 and 5.2, respectively (Table 1).

### Microbial taxonomic diversity based on full-length 16S rRNA genes

**Fig. 1:**
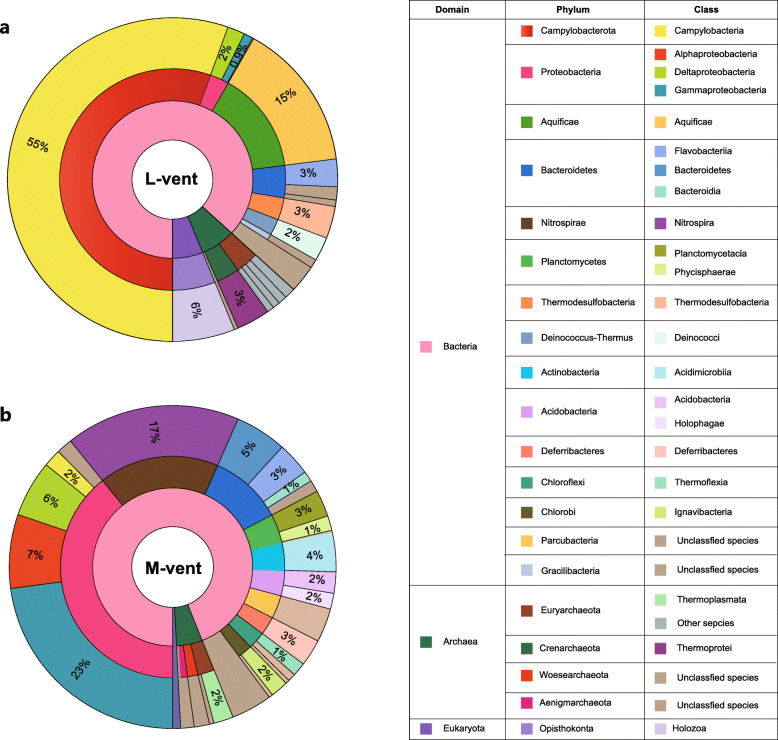
Taxonomic composition of microbial communities from the active L-vent chimney (**a**) and recently inactive M-vent chimney (**b**). Numbers in the pie charts represent percentages of each taxonomic unit in class level, which are estimated based on the full-length 16S rRNA genes retrieved from the two metagenomes. Detailed information is displayed in the Additional file 2: Table S3 and S4

After removing low-quality reads and assembly (general metagenomic information is shown in Additional file 2: Table S2), 162 and 372 full-length 16S rRNA genes were retrieved from the L- and M-vent metagenomes, respectively. The phylogenetic analyses of the 16S rRNA gene showed that the active L-vent chimney was dominated by *Campylobacteria* (phylum *Campylobacterota*) (55.4%), including the genera *Sulfurovum* (20.2%), *Nitratifractor* (8.6%), *Sulfurimonas* (8.6%), and *Caminibacter* (6.3%) (Additional file 2: Table S3). Bacteria belonging to the phylum *Aquificae* had the second highest relative abundance (14.7%), followed by members of the phylum *Chlorobi* (4.7%), *Thermodesulfobacteria* (3.2%), and *Deinococcus*-*Thermus* (2.4%) (Fig. 1a). In contrast, the bacterial community of the inactive M-vent chimney was mainly composed of *Gammaproteobacteria* (22.9%) and *Nitrospirae* (17.3%), as well as *Alpha*- and *Deltaproteobacteria* (7.4% and 5.6%, respectively) (Fig. 1b). The detailed taxonomic information and relative abundance of all reconstructed 16S rRNA genes are listed in Additional file 2: Table S3 and S4.

### Distribution of key metabolic genes

**Fig. 2:**
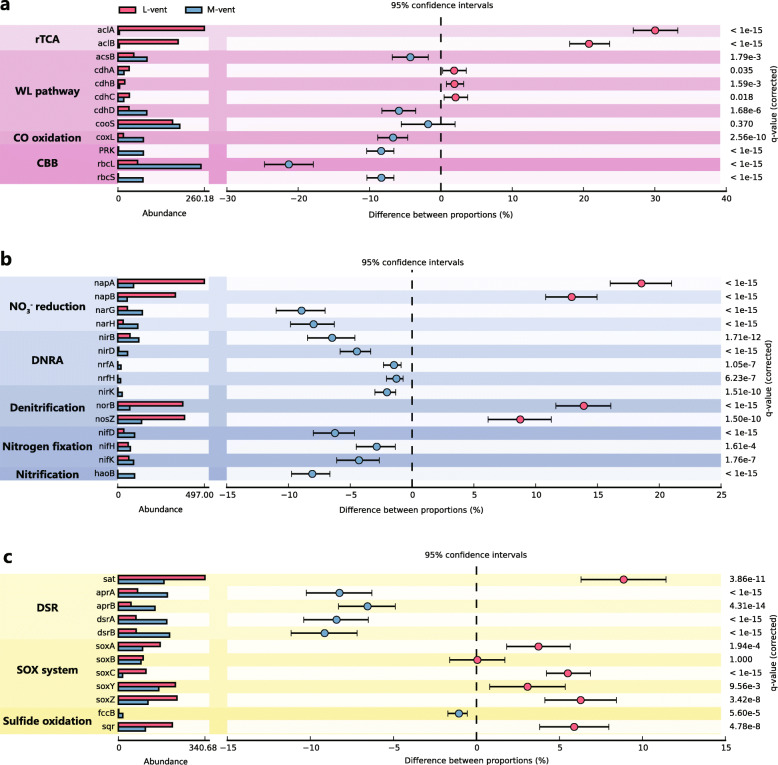
Abundance comparison of key genes involved in carbon, nitrogen, and sulfur metabolisms between active L-vent chimney and recently inactive M-vent chimney. **a** Key genes of rTCA, WL pathway, CBB, and CO oxidation. **b** Key genes of nitrate reduction, DNRA, denitrification, nitrogen fixation, and nitrification. **c** Key genes of DSR, Sox system, and sulfide oxidation. rTCA, reverse citric acid cycle; CBB, Calvin-Benson-Bassham pathway; WL, Wood-Ljungdahl pathway; DNRA, dissimilatory nitrate reduction to ammonia pathway; DSR, dissimilatory sulfate reduction pathway. The *napA/B* and *narG/H* genes encoding nitrate reductase are tested individually and not included in the DNRA and denitrification pathway. The *P* values are based on Fisher’s exact test and corrected by Benjamini-Hochberg FDR

Key genes for microbial carbon, nitrogen, and sulfur metabolisms were searched in the metagenomes of the two chimneys, and differences were revealed regarding gene inventories and the pathways utilized by these two communities (Fig. 2).

#### Carbon fixation

Genes encoding for the ATP-citrate lyase (*aclA/B*), the key enzyme of reductive tricarboxylic acid (rTCA) cycle, were identified in significantly higher abundance (*P* value < 0.05) in the active L-vent sample compared to M-vent (Fig. 2a), and more than 99% of them share high similarities with those from *Campylobacteria* and *Aquificae* (Additional file 1: Figure S1a). In contrast, genes encoding enzymes of the Calvin-Benson-Bassham (CBB) cycle (*rbcL/S* and *PRK*) are significantly enriched in the inactive M-vent chimney, and the majority (43% of *rbcL*; 86% of *rbcS* and 75% of *PRK*) are assigned with *Gammaproteobacteria* (Additional file 1: Figure S1b). For the Wood-Ljungdahl (WL) pathway, genes encoding for the delta subunit of the archaeal acetyl-CoA decarbonylase/synthase complex (*cdhD*) and for the bacterial acetyl-CoA synthase (*acsB*) were more prevalent in the M-vent community, while the genes encoding for the alpha, beta, and epsilon subunits of the archaeal acetyl-CoA decarbonylase/synthase complex (*chdA*, *cdhC*, and *cdhB*, respectively) were present in higher abundances in the L-vent community (Fig. 2a).

#### Nitrogen metabolism

Genes encoding the periplasmic nitrate reductase (*napA/B*) and membrane-bound nitrate reductase (*narG/H*) were identified in both L- and M-vent samples, but with distinctly different abundances (Fig. 2b). In the M-vent chimney, *narG/H* were significantly enriched, with 43% of *narG* assigned to *Alphaproteobacteria* (Additional file 1: Figure S1b), while *napA/B* were more enriched in the active L-vent chimney, with 98% of *napA* assigned to *Campylobacteria* and *Aquificae* (Additional file 1: Figure S1a). Genes of the dissimilatory nitrate reduction to ammonia (DNRA) pathway were more abundant in the inactive M-vent chimney, with 74% of nitrite reductase large subunit (*nirB*) assigned to the *Gammaproteobacteria* (Additional file 1: Figure S1b). For the denitrification pathway, the gene encoding for the beta subunit of the nitric oxide reductase (*norB*) and for the nitrous-oxide reductase (*nosZ*) were identified in significantly higher abundance in the active L-vent chimney compared to M-vent (Fig. 2b), with the majority of them assigned to *Campylobacteria* and *Aquificae* (Additional file 1: Figure S1a; 80% of *norB* and 75% of *nosZ*). On the other hand, the M-vent community was more enriched in genes encoding for subunits of the nitrogenase (*nifD/K/H)*, which is involved in N_2_-fixation, compared to L-vent (Fig. 2b), with 43% of *nifH* being assigned to *Nitrospirae* (Additional file 1: Figure S1b).

#### Sulfur metabolism

A significantly higher abundance of genes encoding for adenylylsulfate reductase (*aprA/B*) and sulfite reductase (*dsrA/B*) were identified in the M-vent sample (Fig. 2c). Particularly, most *aprA/B* were taxonomically assigned to *Gamma*- and *Deltaproteobacteria* (Additional file 1: Figure S1b; 60% of *aprA* and 68% of *aprB*). Since the majority of *dsrA/B* were assigned to unclassified species, we inferred the taxonomy and catalytic type of *dsrA* based on their phylogenies. The results suggest that 13 of 14 *dsrA* presented in the L-vent were of the reductive type, including *Deltaproteobacteria*, *Archaeoglobus*, and *Acidobacteria*, while 36 of 72 *dsrA* genes from M-vent were of the oxidative type belonging to sulfur-oxidizing *Alpha*- and *Gammaproteobacteria*, with the remainder being of the reductive type belonging to *Deltaproteobacteria* (10), *Nitrospirae* (12), and *Acidobacteria* (14) (Additional file 1: Figure S2). For the Sox sulfur oxidation system, similar abundances were found for *soxB* from L- and M-vent; however, the majority of *soxB* from L-vent were assigned to *Aquificae* and *Campylobacteria*, while those from M-vent were largely assigned to *Gamma-* and *Alphaproteobacteria* (Additional file 1: Figure S3). On the other hand, *soxA/C/Y/Z* were found highly enriched in the active L-vent chimney, most of which (95%) were assigned to the *Aquificae* and *Campylobacteria* (Additional file 1: Figure S1a). Additionally, genes encoding for the sulfide-quinone oxidoreductase (*sqr*) were present in higher abundance in the L-vent community, with a similar taxonomic profile as the *sox* genes (Additional file 1: Figure S1a).

### Phylogeny of MAGs

After filtration of low-quality MAGs, 71 and 102 MAGs with a completeness ≥ 70% and potential contamination ≤ 9%, which is higher than the MAG medium quality standard proposed by Genomic Standard Consortium (https://gensc.org/), were obtained for further analysis from L- and M-vent metagenomes, respectively (Additional file 2: Table S5). For L- and M-vent, 42.3% and 48.3% of reads were retrieved to their respective MAGs. Based on the sequencing depth, 20 and 34 of the top 50 most abundant *RpS3* genes were identified in the MAGs recovered from L- and M-vent, respectively, including the top three of the L-vent community and the most and third most abundant taxa of M-vent (Additional file 1: Figure S4). Therefore, the retrieved MAGs are representative of the majority of microbial taxa of both communities.

**Fig. 3:**
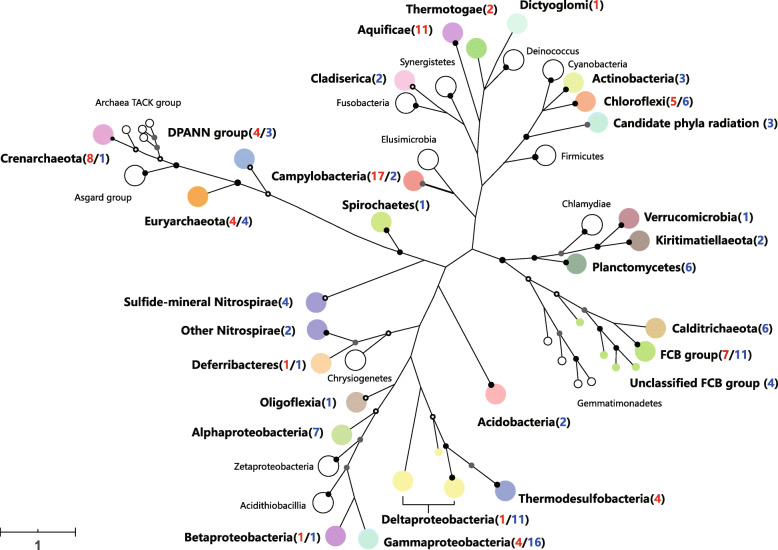
Phylogeny of 173 high-quality MAGs recovered from active L-vent chimney and recently inactive M-vent chimney. The phylogenetic tree is based on 37 concatenated ribosomal proteins and collapsed at the phylum level (class for *Proteobacteria* and *Campylobacteria*). Color nodes represent the MAGs assigned within this clade were retrieved from at least one chimney sample. The red and blue numbers in the parenthesis denote the number of MAGs recovered from L- and M-vent, respectively. Support for internal nodes was constructed from 1000 bootstrap replicates (white ⩾ 50%, gray ⩾ 75%, black ⩾ 95% confidence, no shading ⩽ 50%)

Overall, the 173 retrieved MAGs could be taxonomically assigned to more than 20 phyla, including several novel candidate bacterial phyla without cultivated representatives (Fig. 3). Relative abundance and major groups (> 1%) and individual MAG were shown in Additional file 2: Table S6 and S10. Particularly, for the 71 MAGs from the L-vent chimney, 11 MAGs were taxonomically assigned to the phylum *Aquificae*, which was identified as the dominant taxon (14.5%) based on their reads mapped to the whole L-vent metagenome. Four MAGs of *Thermodesulfobacteria* were the second most abundant bacterial group (8.0%). Seventeen MAGs belonged to *Camplyobacteria* representing only 3.6% of the whole microbial community; this discrepancy to the 16S rRNA gene-based results is probably due to their high interspecies diversity and similar genomic features making it difficult to retrieve more MAGs. *Chloroflexi* (5 MAGs), *FCB* group (7 MAGs), *Gammaproteobacteria* (4 MAGs), and *Thermotogae* (2 MAGs) accounted for 2%, 1.7%, 1.5%, and 1.1%, respectively. For Archaea, 4 and 8 MAGs were assigned to the phyla *Euryarchaeota* and *Crenarchaeota*, representing 4.6% and 2.8%, respectively (Additional file 2: Table S6). Phylogenetic analysis indicated that 3 out of 4 Euryarchaeotal MAGs belonged to the methanogenic classes *Methanococci* and *Methanopyri* and most of the *Crenarchaeota* were distantly related to *Ignicoccus* (Additional file 1: Figure S8). Moreover, 4 MAGs were classified as *DPANN* groups, including *Micrachaeota* (2), *Diapherotrites* (1), and *Nanohaloarchaeota* phyla (1).

For the M-vent sample, 16 *Gammaproteobacteria* MAGs accounted for 11.4% of the whole community, most of which were assigned to *Ca.* Tenderia electrophaga and also closely related to those recovered from previously analyzed inactive chimneys (Additional file 1: Figure S7) [37]. Eleven *Deltaproteobacteria*l MAGs were recovered with a total relative abundance of 4.9%. In addition, *FCB* group (11 MAGs; 4.99%), *Calditrichaeota* (6 MAGs; 3.72%), *Alphaproteobacteria* (7 MAGs; 2.77%), *Nitrospirae* (6 MAGs; 2.94%), *PVC* group (9 MAGs; 4.35%), and *Acidobacteria* (4 MAGs; 2.57%) were represented as major microbial groups among the MAGs of the M-vent microbial community. Phylogenetic analysis showed that the 4 out of the 6 *Nitrospirae* MAGs could be assigned to a newly identified “sulfide-mineral” clade, along with 5 additional MAGs either from inactive sulfide chimneys or subseafloor massive sulfides (SMS) [37, 71] (Fig. 3), while the other 2 *Nitrospirae* MAGs were part of a separate clade (Additional file 1: Figure S6). The genome tree further showed that *Nitrospirae* are split into two distinct lineages with long phylogenetic distance (Fig. 3), in line with the polyphyletic feature of *Nitrospirae* reported before [62], highlighting the need to reclassify this phylum. In addition, 3 MAGs were assigned to novel taxa in the candidate phyla radiation (CPR) and 2 MAGs were assigned to the phylum *Micrachaeota* in *DPANN* group.

### Index of replication value (iRep) of bacterial MAGs

**Fig. 4:**
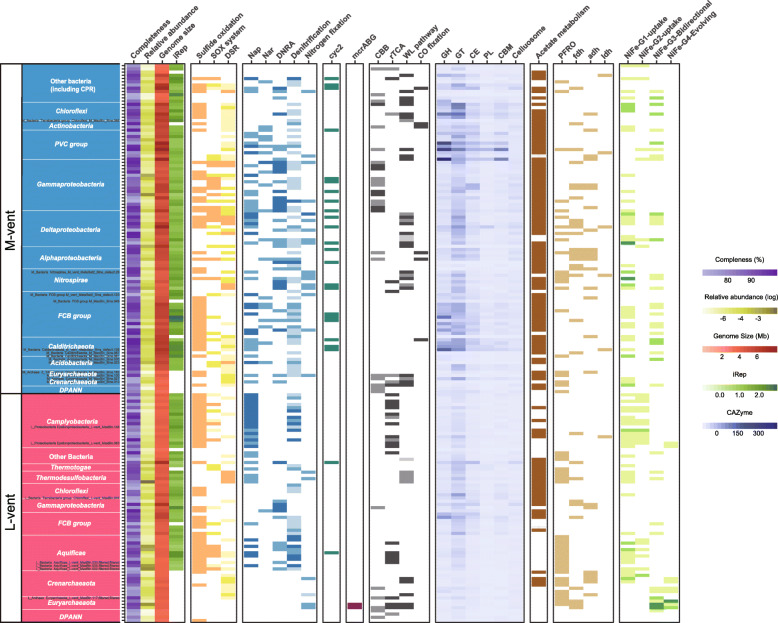
Genomic features and metabolic potential of 173 MAGs retrieved from the active L-vent and recently inactive M-vent chimney. Different color gradients in completeness, relative abundance (logged), genomic size, iRep value, and CAzyme represent their quantities among MAGs. Differentially shaded tiles represent the completeness of displayed metabolic pathways, including none, partial, incomplete, and complete four levels. There are only two levels (encode or not) for *cyc2* and genes involved in fermentation. The specific key genes and completeness definition involved in each metabolic pathway could find in Additional file 2: Table S7

The iRep value provides information about the replication activity of specific MAGs at the time of sampling (see the “Material and methods” section). Theoretically, a iRep value of 1.5 means that half of the cells in a population are replicating, but in reality, there are several ways to achieve a given iRep value since the population is heterogeneous, i.e., some cells may not replicate and others are replicating at a faster rate with more than one replication fork [67]. In this study, most of the retrieved bacterial MAGs in the chimneys represent active replicating bacterial taxa as indicated by iRep values calculated from 52 and 91 high-quality bacterial MAGs from the L- and M-vent samples, respectively. The average iRep value of bacterial MAGs from the inactive M-vent is 1.51, which is higher than that from the active L-vent (1.42) (Additional file 2: Table S6). In the L-vent, *Camplyobacteria* had the highest average iRep value (1.52), followed by *Chloroflexi* (1.5), *Gammaproteobacteria* (1.47), *Thermodesulfobacteria* (1.43), and *Aquificae* (1.40). In the M-vent, *Calditrichaeota* had the highest iRep value (1.8), followed by the *FCB* group (1.74), *Chloroflexi* (1.59), *Nitrospirae* (1.49), *Alpha-*/*Deltaproteobacteria* (1.48 and 1.42, respectively), and *Gammaproteobacteria* (1.4). iRep values for each MAG and the average iRep value of other major microbial groups (> 1% in each sample) are shown in the Fig. 4 and Additional file 2: Table S6, respectively.

### Metabolic reconstruction of MAGs

#### The active L-vent chimney

Based on the analysis of MAGs, the *Campylobacteria* (17 MAGs) and *Aquificae* (11 MAGs) dominating the active L-vent are potential sulfur/hydrogen-oxidizing bacteria with capabilities of denitrification and carbon fixation through the rTCA cycle (Fig. 4). All 28 MAGs encode at least one *sqr* gene; 45% and 24% of them also encode complete or near complete Sox system (Fig. 4; Additional file 2: Table S6). The rTCA cycle is the sole carbon fixation pathway and was prevalently identified in the *Aquificae* and *Campylobacteria* MAGs (55% and 47%, respectively). In addition, the majority of *Aquificae* and *Campylobacteria* MAGs encode hydrogenase groups 1 and 2 for hydrogen uptake. Further, *napA/B* genes were identified in every MAG assigned to *Campylobacteria* and 45% of *Aquificae* MAGs*.* Genes encoding for the enzymes catalyzing the subsequent steps of denitrification (*nirS/K*, *norB/C*, *nosZ*; Additional file 2: Table S7) were identified in 73% and 47% of the MAGs belonging to the *Aquificae* and *Campylobacteria*, respectively (Fig. 4; Additional file 2: Table S6).

Besides *Aquificae* and *Campylobacteria*, 4 MAGs (8.2%) belonging to the *Thermodesulfobacteria* were identified in the L-vent chimney that have the capacity of reducing multiple sulfur species. They contained not only the key genes encoding the complete sulfate reducing pathway (i.e., *sat*, *aprA/B* and *dsrA/B*), but also other essential marker genes like *dsrD*, the sulfite reductase-associated electron transfer complex (*dsrM/K/J/O/P*), and the electron transfer complex (*QmoA/B/C*) (Additional file 2: Table S8). Moreover, genes encoding for the thiosulfate reductase (*phsA/B*) and tetrathionate reductase (*ttrA*) were also identified in 2 of them. Thermodesulfobacteria MAGs from L-vent chimney share highly similar metabolic potential with their sulfur-disproportioning isolates [72].

We also identified 2 MAGs belonging to the phylum *Euryarchaeota* that contained the complete gene cluster encoding for the methyl coenzyme M reductase *mcrABG* and also genes encoding for the Group 3/4 hydrogenase, indicating a methanogenic metabolism (Fig. 4; Additional file 2: Table S6). The other major microbial groups, such as *Chloroflexi* and the *FCB* group, have organotrophic potential, either using fermentation or respiration, which is supported by the considerable number of genes related to carbohydrate degradation and nitrate reduction (Fig. 4).

#### The recently extinct M-vent chimney

Based on the analysis of MAGs, the *Gammaproteobacteria* (16 MAGs) dominating the M-vent chimney are potential chemoautotrophic sulfur-oxidizing bacteria, using the CBB cycle for carbon fixation and reducing nitrate via the DNRA pathway (Fig. 4). For sulfur oxidation, most of the MAGs contained the genes encoding for the Sox system (69%) and *sqr* gene (63%) (Fig. 4; Additional file 2: Table S6). In addition, 50% of the MAGs also contain the gene encoding for the reverse DSR as evidenced by the phylogenetic assignment of the *dsrA* gene (Additional file 1: Figure S2), indicating the potential for the oxidative DSR pathway for sulfur oxidation. Moreover, 5 of these MAGs also contain the *cyc2* gene encoding for an outer membrane *c*-type cytochrome, which is closely related with their expressed homologs in the electroautotrophic *Ca.* Tenderia electrophaga (Additional file 1: Figure S9).

*Deltaproteobacteria* (11 MAGs) were identified as one of the major microbial taxa in the M-vent chimney. Based on their gene content, they are putative sulfate-reducing bacteria (SRB) having the potential to oxidize organic matter through the WL pathway, with 64% of the MAGs encoding the reductive DSR pathway and WL pathway. Specifically, genes involved in carbohydrate degradation are significantly enriched in the *Deltaproteobacteria* (19.2 CAZyme genes per MAG on average; Additional file 2: Table S6). Based on the identification of genes encoding for *napAB/narGH* and the subsequent DNRA pathway in most of their MAGs, nitrate appears to be a potential alternative electron acceptor for *Deltaproteobacteria*.

The 4 *Nitrospirae* MAGs recovered belonging to the “sulfide-mineral” clade encode essential genes of DSR, WL pathway, nitrite reduction and nitrogen fixation, same as the other 5 MAGs in this clade (Fig. 4; Additional file 2: Table S9). Three of them encode the key enzyme of *cbb3*-type cytochrome c oxidase (Additional file 2: Table S10). Furthermore, 5 of all 9 *Nitrospirae* from the “sulfide mineral” clade (including the 2 most abundant recovered from the M-vent and the other 3 derived from recently extinct sulfide chimneys and SMS, respectively [37, 71]) encode the *cyc2* gene (Additional file 2: Table S9). Their *cyc2* genes are phylogenetically closely related to each other and distantly related to their counterparts identified in the genomes of *Zeta*-/*Betaproteobacteria* Fe-oxidizing bacteria (FeOB) [73] (Additional file 1: Figure S9). Moreover, these “sulfide mineral” *Nitrospirae* MAGs have relatively small genomes (< 2 Mb) and fewer genes involved in sulfur oxidation pathways (*sqr* and *sox*) compared with other *Nitrospirae* species (Additional file 2: Table S9). Besides the 4 MAGs assigned to the “sulfide mineral” clade, the other two *Nitrospirae* MAGs recovered from the M-vent have distinct metabolic features: one does not have any genes involved in sulfate reduction; the other one encodes the rTCA pathway instead of the WL pathway and is closely related with another metabolically similar *Nitrospirae* MAG recovered from a long-time inactive sulfide chimney [37] (Additional file 2: Table S9).

Based on the prevalence of genes encoding for *narGH*, *napAB*, and the subsequent DNRA pathway (*nrfA/H*, *nirB*, and *nirD*; Additional file 2: Table S7) in their genomes, other microorganisms in the M-vent chimney including the *FCB* group, *PVC* group, *Calditrichaeota*, *Alphaproteobacteria*, *Chloroflexi*, and *Actinobacteria* are likely to be nitrate-respiring heterotrophs (Fig. 4). That is also supported by the enrichment of genes for carbohydrate degradation (CAZyme) identified in their MAGs, especially for the *Calditrichaeota*, *FCB*, and *PVC* group (Fig. 4). Interestingly, some *Calditrichaeota* (1 in 6 MAGs), *Alphaproteobacteria* (2 in 7 MAGs), and *Actinobacteria* (2 in 3 MAGs) encode carbon monoxide dehydrogenase (*coxM/L/S*) which catalyzes CO oxidation. The phylogenetic analysis of *coxL* from M-vent suggests that they are largely assigned to the putative FormII/BMS clade (Additional file 1: Figure S5). The *Euryarchaeota* MAGs recovered from M-vent are potential sulfate reducing archaea that are phylogenetically closely related to the *Archaeoglobi* lineage, which is supported by the retrieved 16S rRNA gene of the genus *Geoglobus* (Additional file 2: Table S4). Three of the 4 MAGs encode the complete reductive DSR pathway, the archaeal WL pathway, as well as group 1 hydrogenase (Fig. 4; Additional file 2: Table S6).

## Discussion

**Fig. 5:**
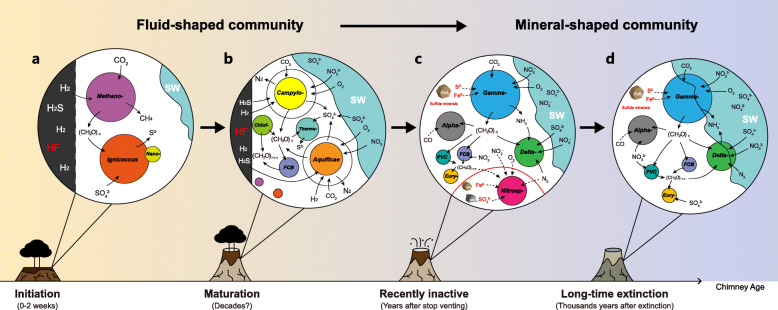
Genome-resolved conceptual models of microbial succession within the sulfide chimney from initiation, maturation, recently inactive, and long-time extinction. The models of maturation and recently inactive stage are based on L-vent and M-vent in this study; data of initiation and long-time extinction stage are summarized from previous studies. HF, hydrothermal fluids; SW, seawater; Methano-, *Methanocaldococcus*; Nano-, *Nanoarchaeum*; Campylo-, *Campylobacteria*; Chlof-, *Chloroflexi*; Thermo-, *Thermodesulfobacteria*; FCB, *FCB* group; Alpha-, *Alphaproteobacteria*; *Gamma*-, *Gammaproteobacteria*; Delta-, *Deltaproteobacteria*, Nitrosp-, *Nitrospirae*; PVC, *PVC* group; Eury-, *Euryarchaeota*; FeS_2_, pyrite-like sulfide minerals; CaSO_4_, anhydrite-like sulfate minerals

Microbial communities inhabiting hydrothermal sulfide chimneys are largely shaped by the local geochemical, physical, and geological conditions, and consequently, the taxonomy and metabolic capabilities vary with the development of sulfide chimneys [33–37]. Due to the unpredictable nature of volcanic eruptions combined with sampling challenges, it has proven difficult to follow and investigate the succession of microbial communities of a sulfide chimney from its creation to extinction. Here, we present the first reported metagenome from an inactive sulfide chimney that went extinct at a precisely known time after a volcanic eruption in 2006, making it significant to understand the microbial succession taking place during the initial transition from an active chimney to an inactive one. Moreover, we also retrieved and deciphered 173 high quality MAGs from both chimneys, which provide genomic insights into the metabolic functions and ecological roles of these microbes. Combining data obtained in the present study with those published previously (including studies from Guaymas Basin, East Pacific Rise 9°/13° N, Mid-Atlantic Ridge, Main Endeavor Field, Loki’s Castle vent field; as well as some in situ incubations and lab enrichments, details see Additional file 2: Table S11), we are proposing a conceptual model to understand the pattern of microbial succession: microbes in the sulfide chimney shift from a “fluid-shaped” community supported by electron donors in the fluid into a “mineral-shaped” one supported by solid-phase electron donors during the lifetime of a vent from initiation to extinction (Fig. 5). Enrichments in Ca^2+^ and K^+^ and depletions in Mg^2+^ and SO^2−^_4_ relative to seawater in the M-vent fluid (Table 1) are consistent with its composition being regulated by subsurface fluid-rock reactions similar to those responsible for the formation high-temperature (300–400 °C) fluids venting in the vicinity of 9° 50′ N EPR. Measured Mg^2+^ and SO^2-^_4_ concentrations, however, did not approach zero as is typically observed for high-temperature vent fluids and were accompanied by a temperature of only 35 °C, indicating mixing with cold seawater and conductive cooling had occurred in subseafloor environments. In response to abiotic and biotic processes following subsurface mixing and cooling, the M-vent fluids are highly depleted in dissolved H_2_ and H_2_S relative to what would be expected for conservative mixing of a high-temperature (~ 350 °C) endmember fluid and seawater. Consequently, H_2_ and H_2_S, two key electron donors that participate in a variety of metabolic processes, have extremely limited availability to support chimney ecosystems. In contrast to M-vent, the L-vent fluids have substantially higher measured temperatures and deliver abundant H_2_ and H_2_S to chimney environments at levels typical of high-temperature hot springs at EPR.

### The “fluid-shaped” microbial community

A few studies on the microbial pioneers colonizing a nascent chimney are all based on in situ or simulated incubation experiments, suggesting that microbes colonize the initially sterile sulfide structures quickly, and get established within a short period of time (< 14 days) [74–79]. Hyperthermophiles, including *Methanocaldococcus* and *Ignicoccus* species together with its symbiont *Nanoarchaeum*, were identified as the dominant pioneer microorganisms of freshly formed chimneys [77, 78, 80]. In the present study, these putative archaeal pioneers were also identified in the active L-vent chimney, including one *Methanocaldococcus*-like MAG in considerable abundance (3.8%; Fig. 4). Metabolic reconstruction suggests that these uncultured organisms have the ability to utilize H_2_ as an electron donor for methanogenesis (*Methanocaldococcus*) and sulfur reduction (*Ignicoccus*), in line with the metabolism of their cultured hyperthermophilic deep-sea vent relatives *Methanocaldococcus* and *Ignicoccus* (Fig. 4) [81, 82]. In view of their requirement for H_2_, high temperature, and anoxic conditions, these hyperthermophilic archaea are likely present in the interior layers of the chimney, where these pioneers are expected to consistently contribute to the primary production throughout the lifetime of an active chimney [83, 84] (Fig. 5a).

During the development of a sulfide chimney, available spatial, redox, and thermal gradients for microorganisms are expanding with accumulating mineral deposits along with the oxidative weathering from seawater. Colonization is followed by the thermophilic *Aquificae* and thermophilic and mesophilic *Campylobacteria*, which colonize the relative cooler exterior layers of the chimney wall and often form massive biomats covering the chimney exterior [20, 21]. For example, the *Campylobacteria* quickly became the dominant community members after 5 days inside the growth chamber of an in situ incubation device [79], sharing high similarity with the taxonomic profile of most investigated mature sulfide chimneys, including L-vent [16–19, 22, 23, 85, 86]. Undoubtedly, H_2_ from venting fluids is the most important electron donor during the early microbial succession stage, not only for archaeal pioneers, but also for the following *Aquificae* and *Campylobacteria* in the mature stage (Fig. 4). Subsequently, oxidation of H_2_S and other reduced sulfide compounds contained in the fluid become important energy sources, as evidenced by the prevalence of genes involved in sulfur oxidation, like *sqr* and the Sox system, identified in most MAGs assigned to these and other bacterial chemolithotrophs (Figs. 2 and 4). Both H_2_S and H_2_ are abundantly available in the vent fluids at L-vent to support chemoautotrophs in the chimney wall (Table 1) In addition, the potentials for aerobic respiration and denitrification indicate that the *Aquificae* and *Campylobacteria* are able to utilize oxygen and nitrate from seawater percolating through the chimney as electron acceptors, further implying that they inhabit the relative outer layers and exterior of the chimney wall. In short, hydrothermal fluid chemistry largely controls the primary microbial colonization in newly formed chimneys and shapes the microbial community within sulfide chimneys from the initial to the mature stages (Fig. 5a, b).

### The “mineral-shaped” microbial community

During the transition phase from a mature to an inactive sulfide chimney, a “fluid shaped” microbiome as described above is expected to shift to a “mineral-shaped” community (Fig. 5c, d). First, the decrease in temperature plays a key role in shaping the microbial community, changing from one dominated by thermophiles to one dominated by mesophiles and finally psychrophiles. This is well supported by the distinct differences between active and inactive sulfide chimneys extinct for > 1000 years, i.e., a community dominated by the *Aquificae* and *Campylobacteria* on one hand and an assemblage of the *Gamma*-/*Delta*-/*Alphaproteobacteria* and *Bacteroidetes* on the other hand [4, 33–35, 84]. Although the M-vent chimney went extinct only 7 years prior to sampling, the overall microbial composition was highly similar to those chimneys that had been extinct for much longer time periods [33, 35–37]. This suggests that the microbial succession takes mainly place during the early stages after venting ceases and that the microbial community then stays relatively stable over long periods of time of up to thousands of years.

Along with the diminishing hydrothermal fluids, available energy sources for chemoautotrophs supporting the DSHV ecosystem gradually shift from reduced chemicals contained in the vent fluids (mainly H_2_ and H_2_S) to the sulfide minerals making up the chimneys. Besides temperature, this is another critical factor in driving microbial succession at the taxonomic and metabolic level once venting ceases. First of all, the *Gammaproteobacteria* are inferred to replace the *Campylobacteria* and *Aquificae* as the major primary producers during this process, instead fixing CO_2_ via CBB cycle and retrieving energy from mineral sulfides through multiple sulfur oxidation pathways (reverse DSR, Sox system and *sqr*). The prevalence of the *cyc2* gene identified in their MAGs suggests that, besides sulfide, Fe^2+^ from minerals (like pyrite and pyrrhotite) likely serves as an alternative electron donor for these autotrophs. This gene encodes an outer membrane cytochrome c, which was demonstrated to mediate iron oxidation in the acidophilic FeOB *Acidithiobacillus ferrooxidans* and has also been found in all available genomes of neutrophilic FeOB and has been proposed as a candidate genetic marker for FeOB [73, 87, 88]. Moreover, a highly expressed *cyc2* gene identified in the electroautotrophic *Ca.* Tenderia electrophaga, which is phylogenetically closely related to the *cyc2* genes identified in the MAGs (Additional file 1: Figure S9), has been inferred to be involved in extracellular electron transfer (EET) [89]. Therefore, this suggests that the *Gammaproteobacteria* identified in M-vent have the potential to use EET to oxidize Fe^2+^ contained in sulfide minerals, like pyrite and pyrrhotite, that make up the chimney structure. In support of this hypothesis, *Thiomicrospira* sp. SC-1, a FeOB recently isolated from an in situ incubation with pyrrhotite, has been shown to grow autotrophically with iron oxides and sulfur intermediates [30, 90]. Thus, by utilizing the oxidation of metal sulfides as an energy source, these putative chemolithoautotrophic *Gammaproteobacteria* very likely play essential roles in supporting the ecosystem of inactive sulfide chimneys for thousands of years after cessation of venting [33–36], similar to the role of *Aquificae* and *Campylobacteria* in active chimneys. Besides the *Gammaproteobacteria*, *cyc2* genes were also identified among many other bacterial taxa in the inactive M-vent chimney, including *Alpha*-/*Deltaproteobacteria*, *Nitrospirae*, and *FCB* group (Fig. 4; Additional file 1: Figure S9). Previously, *cyc2-*containing chemolithotrophic *Alpha*-/*Betaproteobacteria* have been shown to accelerate aerobic pyrite oxidation in freshwater sediments and *Alphaproteobacteria* were also identified in pyrrhotite incubation experiments as dominant members [90, 91]. Thus, our results imply that the capability to oxidize iron sulfides for chemolithotrophic growth may be widespread among microbes living in inactive chimneys.

Generally, putative heterotrophic bacteria dominating the M-vent and other inactive chimneys, like *Alpha*-/*Deltaproteobacteria*, *Chloroflexi*, *PVC*, and *FCB* group, have also been widely identified in active chimneys, although generally with much lower relative abundances [16, 18, 85, 92–94]. Thus, the increasing abundance of heterotrophs in inactive chimneys suggests that they probably already colonized the exterior of actively venting chimneys, and then gradually became more prevalent with diminishing hydrothermal activity. The iRep values indicate that these heterotrophic bacteria are actively replicating in situ, which is consistent with the previous observation of significant enzymatic activity in inactive chimneys [34]. Prevalent cytochrome c oxidase genes identified in most of MAGs from the M-vent suggest that oxygen is very likely a widespread electron acceptor for microbial communities inhabiting inactive sulfide chimneys (Additional file 2: Table S10). Interestingly, compared with the active L-vent chimney, our results further revealed that the metabolic potential for sulfate reduction was more prevalent in the M-vent chimney, both at the gene and genomic level (Figs. 2 and 4). This suggests that sulfate might be an important electron acceptor for the majority of microorganisms in the M-vent chimney. In view of the fact that membrane-bound nitrate reductase encoded by *nar* is more efficient than the *nap*-encoded periplasmic nitrate reductase at high nitrate concentrations [95], the higher frequency of *nar* gene observed in the M-vent possibly reflects the microbial adaption to the increased accessibility of nitrate, implying that the intrusion of seawater plays an important role in the microbial succession after venting ceases.

Based on 16S rRNA gene analysis, *Nitrospirae* was one of the major taxa (17.3%) identified in the recently extinct M-vent chimney (Fig. 1), in contrast to previous studies that have rarely reported this group as one of the dominant taxa in either active or extinct sulfide chimneys [16, 22, 76, 85, 93, 96]. The only exceptions besides the present study are two recently described inactive chimneys, one of them being a relatively young inactive chimney (0 ± 160 years) and the other one a much older inactive chimney (~ 2093 years) with *Nitrospirae* making up 83% and 38% of the community, respectively [37]. Furthermore, the 7-year old inactive M-vent chimney and the younger inactive chimney described by Meier et al [37] share similar dominant *Nitrospirae* phylotypes within a unique “sulfide-mineral” clade that is distant from the *Nitrospirae*-1 phylotype dominating in an older inactive chimney as well as other *Nitrospirae* lineages [37] (Additional file 1: Figure S6). This suggests that the *Nitrospirae* “sulfide-mineral” clade flourishes in the early stage of inactive sulfide chimneys, making them a potential marker microorganism for young, recently extinct sulfide chimneys. Meier et al [37] propose that mineral sulfate, like anhydrite and/or barite, serves as a potential electron acceptor for *Nitrospirae* inhabiting young inactive chimneys [37]. While our data support this hypothesis, the finding of *cyc2* genes in the “sulfide-mineral” clade further suggests that the oxidation of Fe^2+^ might also play an important role for these unique *Nitrospirae* enabling them to thrive in young inactive chimneys. Given that the *cyc2* gene has been described to be involved in the oxidation of external electron donors, such as iron [73, 87, 89], the transferred electrons are very likely coming from the metal sulfides making up inactive sulfide chimneys. In addition, a *Nitrospirae* MAG belonging to the “sulfide mineral” clade was previously recovered from a subsurface massive sulfide (SMS) deposit in the Southern Mariana Trough [71] (Additional file 1: Figure S6), suggesting that there are common environmental features between recently inactive chimneys and SMS, such as the preservation of metal sulfide minerals that are not yet fully oxidatively weathered by permeating seawater. In addition, genes encoding cbb3-type cytochrome c and nitrite reductase were identified in most *Nitrospirae* MAGs belonging to the “sulfide-mineral” clade (Additional file 2: Table S9 and S10), implying that oxygen and nitrite might be potential electron acceptors for conserving energy via chemolithotrophic iron oxidation (Fig. 5). Thus, sulfide mineral utilization might be a key factor allowing these *Nitrospirae* to thrive in recently inactive sulfide chimneys, whose decay by oxidative weathering is largely proportional to the age of the inactive chimney, but is also controlled by their particular mineralogical and geological properties.

## Conclusions

Here, the metagenome of a sulfide chimney that became recently inactive (~ 7 years prior to sampling) is being compared with an actively venting chimney from the same hydrothermal vent field located on the East Pacific Rise at 9–10° N. Their microbial communities have distinct compositional structures and energy-yielding metabolic potentials, indicating that the changes of the microbial community are largely driven by the available energy sources that shift from venting fluids to the mineral phase as the hydrothermal activity diminishes. Based on the results presented here, the transition from a “fluid-shaped” to a “mineral-shaped” community occurs within years after venting ceases, after which the communities stay stable for thousands of years. We could further identify a unique “sulfide-mineral” *Nitrospirae* clade, making it a potential marker microorganism for young, recently extinct sulfide chimneys. More sampling and further experiments are needed to elucidate the specific ecological significance of this clade during the succession from active to inactive chimneys, as well as the mechanisms involved in oxidation of sulfide minerals and the utilization of sulfate minerals as an electron acceptor.

## Supplementary information

**Additional file 1: Figure S1.** Taxonomic classification of key functional genes retrieved from the L- and M-vent chimney. (a) The key genes enriched in the active L-vent chimney. (b) The key genes enriched in the recently inactive M-vent chimney. **Figure S2** Maximum-likelihood phylogeny of *dsrA* genes retrieved from L-vent and M-vent chimney. The red branches represent the *dsrA* genes recovered from active the L-vent chimney, while the blue ones are those from recently inactive M-vent chimney. Numbers of *dsrA* gene for each sample are displayed in the parenthesis after the clade name. **Figure S3** Maximum-likelihood phylogeny of *soxB* genes retrieved from L-vent and M-vent chimney. The red nodes represent the *soxB* genes recovered from the active L-vent chimney, while the blue ones are those from the recently inactive M-vent chimney. Four *soxB* genes from M-vent chimney form a distinct clade (marked with red star) that different with other *soxB*. **Figure S4** Distribution and quantity of top 50 *RpS3* genes retrieved in the MAGs for two chimney samples. (a) in the active L-vent chimney. (b) in the recently inactive M-vent chimney. The x-axis represents each *RpS3* gene identified in two samples, the y-axis is the sequencing depth for each *RpS3* gene. The red bars indicate the *RpS3* genes identified in the MAGs in corresponding samples, while the blue ones are those are not included in the MAGs. **Figure S5** Maximum-likelihood phylogeny of *coxL* genes retrieved from L- and M-vent chimney. The red branches represent the *coxL* genes recovered from active the L-vent chimney, while the blue ones are those from recently inactive M-vent chimney. Support for internal nodes was constructed from 1000 bootstrap replicates, black dots represent those nodes with bootstrap value > 75%, which is direct proportional to their diameter. **Figure S6** Phylogeny of 6 *Nitrospirae* MAGs recovered from M-vent chimney. The phylogenetic tree is based on 37 concatenated ribosomal proteins and collapsed at the genus level. The red nodes represent those MAGs recovered from M-vent chimney, the green ones are those from subseafloor massive sulfide deposits [71] and the organe ones are those retrieved from inactive chimneys [37]. The green-shaded branches are proposed as “Sulfide mineral” clade of *Nitrospirae* in this study. **Figure S7** Phylogeny of 20 *Gammaproteobacteria* MAGs recovered from M- and L-vent chimney. The phylogenetic tree is based on 37 concatenated ribosomal proteins and collapsed at the genus level. The red nodes represent those MAGs recovered from L-vent chimney, and blue for M-vent chimney. The organe ones are those retrieved from inactive chimneys [37]. **Figure S8** Phylogeny of 8 *Crenarchaeota* MAGs recovered from L-vent chimney. The phylogenetic tree is based on 37 concatenated ribosomal proteins and collapsed at the family level. The red nodes represent those MAGs recovered from L-vent chimney. **Figure S9** Maximum-likelihood phylogeny of *cyc2* genes retrieved from L- and M-vent chimney. The red branches represent the *cyc2* genes recovered from the active L-vent chimney, while the blue ones are those from recently inactive M-vent chimney. the green ones are those from subseafloor massive sulfide deposits [71] and the organe ones are those retrieved from inactive chimneys [37]. The other reference sequences are come from Kato et al., 2015 [71]. Support for internal nodes was constructed from 1000 bootstrap replicates, black dots represent those nodes with bootstrap value > 70%, which is direct proportional to their diameter.

**Additional file 2: Table S1** Measured concentrations of selected aqueous species and pH in replicate fluid samples from M-vent and L-vent. **Table S2** General metagenomic and assembly characteristics of M- and L-vent chimney sample. **Table S3** Taxonomic classification and relative abundance of full-length 16S rRNA genes recovered from L-vent sulfide chimney sample. **Table S4** Taxonomic classification and relative abundance of full-length 16S rRNA genes recovered from M-vent sulfide chimney sample. **Table S5** Completeness and contamination of 173 MAGs recovered from L- and M-vent chimney samples. **Table S6**: Metabolic potential of MAGs for the major microbial groups (> 1%) recovered from the L- and M-vent chimney. The specific method for estimating relative abundance/iRep are listed in the "Method and Materials" in the main text. The average iRep value of bacterial MAGs recovered from L- and M-vent sample are 1.42 and 1.51, respectively. The numbers in the metabolic pathway columns represent the percentage of the microbial taxa encoded the complete pathway (based on quantity of MAGs, e.g. 0.33 means 1 in 3 of the MAGs have the pathway). **Table S7** Key genes of metabolic pathways for MAG annotation. The completeness of each metabolic pathway is classified into 3 level: 1- complete, 2 - uncomplete; 3- partial. The particular completeness classification standard for each pathway are listed in the following: Sulfide oxdation: complete-sqr or fccB; Sox pathway (thiosulfate oxidation): complete - *sox A/B/X/Y/Z/C/D*, uncomplete - *soxB*, partial: other Sox genes expect *soxB/C/D*; Dissimilatory sulfate reduction/oxidation: complete-*sat/dsrAB/aprAB*, uncomplete - two of *sat/dsrAB/aprAB*, partial - one of *sat/dsrAB/aprAB*. Subsequent dissimilatory nitrate reduction: complete-*nirB* or *nrfA/H*; uncomplete-one of *nrfA/H*. Subsequent denitrification: complete-*nirS/K* and *norB/C* and *nosZ*; uncomplete - two of *nirS/K* and *norB/C* and *nosZ*; partial - one of *nirS/K* and *norB/C* and *nosZ*. Nitrogen fixation: complete-*nifK/D/H*; uncomplete - two of *nifD/H/K*; partial - one of *nifD/K/H*. WL pathway: complete-*cdhC* or *acsB* and *cdhE* and *cdhD* and *cooS*; *cdhC* or *acsB* and one of *cdhE/D* and *cooS*; partial - two of *cdhC/B* and *cdhE/D* and *cooS.* CO oxidation: complete - *coxM/L/S*; uncomplete - *coxL* and one of *coxS/M*; partial - one of *coxM/L/S*. Acetate utilization/production: complete - *ACSS* or *pta/ack*. rTCA: complete - *aclA/B*; uncomplete - *aclA* or *aclB*. CBB pathway: complete - *rbcL/S*; uncomplete - *rbcL* or *rbcS*. Methanogenesis/aerobic methane oxidation: complete - *mcrA/G/B*; uncomplete: two of *mcrA/B/G*; partial - one of *mcrA/G/B*. Cytochrome c oxidase: complete - two *Cox/Cyd/Qox* genes or three *cco/Cyo* genes; uncomplete- one of *Cox/Cyd/Qox* genes or two of *cco/Cyo* genes; partial- one of *cco/Cyo* genes. **Table S8** Sulfur metabolic pathways of *Thermodesulfobacteria* MAGs recovered. **Table S9** Metabolic potential of *Nitrospirae* MAGs retrieved from this study and phylogenetically closed species. The red ID represented those *Nitrospirae* MAGs reconstructed from M-vent, the green ones represent those recovered from subseafloor massive sulfides [71] and the blue ones refer to those *Nitrospirae* retrieved from inactive sulfide chimneys [37]. Numbers in the form represent the completeness of specific pathways for each *Nitrospirae* genome/MAG, 1 indicates that complete key genes of the pathway were identified in the genomes/MAGs, other else the specific key genes were listed if the pathway is not complete. For *sqr*, cyctochrome C oxidase, multi-heme cyc and *cyc2*, numbers represent the quantity of specific genes identified in the genomes/MAGs. **Table S10** Distribution and quantity of cytochrome c oxidase genes in 173 MAGs retrieved from M- and L-vent. Specific definition rules and types of cytochrome c oxidase genes could find in Table S7. **Table S11** Summary of the activity, study method and microbial composition of deep-sea hydrothermal sulfide chimneys or in situ incubation experiments cited in this study.

## Data Availability

Metagenomic-assembled sequences are available in the Integrated Microbial Genomes and Microbiomes (IMG/M) database with IMG Object ID 3300005095/3300005096. All MAGs from the current study have been deposited in the NCBI GenBank under the project ID PRJNA557557.

## References

[1] Francheteau J, Needham H, Choukroune P, Juteau T, Seguret M, Ballard RD, Fox P, Normark W, Carranza A, Cordoba D (1979). Massive deep-sea sulphide ore deposits discovered on the East Pacific Rise. Nature.

[2] Dick GJ (2019). The microbiomes of deep-sea hydrothermal vents: distributed globally, shaped locally. Nat Rev Microbiol.

[3] Lowell RP, Seewald JS, Metaxas A, Perfit MR. Magma to microbe: modeling hydrothermal processes at oceanic spreading centers: John Wiley & Sons; 2013.

[4] Kelley DS, Baross JA, Delaney JR (2002). Volcanoes, fluids, and life at mid-ocean ridge spreading centers. Annual Review of Earth and Planetary Sciences.

[5] Fornari D, Von Damm K, Bryce J, Cowen J, Ferrini V, Fundis A, Lilley M, Luther G, Mullineaux L, Perfit M (2012). The East Pacific Rise between 9°N and 10°N: twenty-five years of integrated, multidisciplinary oceanic spreading center studies. Oceanography.

[6] Tolstoy M, Cowen JP, Baker ET, Fornari DJ, Rubin KH, Shank TM, Waldhauser F, Bohnenstiehl DR, Forsyth DW, Holmes RC (2006). A sea-floor spreading event captured by seismometers. Science.

[7] Haymon R, Fornari D, Von Damm K, Lilley M, Perfit M, Edmond J, Shanks W, Lutz R, Grebmeier J, Carbotte S (1993). Volcanic eruption of the mid-ocean ridge along the East Pacific Rise crest at 9 45–52′ N: Direct submersible observations of seafloor phenomena associated with an eruption event in April, 1991. Earth and Planetary Science Letters.

[8] Tan YJ, Tolstoy M, Waldhauser F, Wilcock WS (2016). Dynamics of a seafloor-spreading episode at the East Pacific Rise. Nature.

[9] Soule SA, Fornari DJ, Perfit MR, Rubin KH (2007). New insights into mid-ocean ridge volcanic processes from the 2005–2006 eruption of the East Pacific Rise, 9 46′ N–9 56′ N. Geology.

[10] Kristall B, Kelley DS, Hannington MD, Delaney JR: Growth history of a diffusely venting sulfide structure from the Juan de Fuca Ridge: a petrological and geochemical study. Geochemistry, Geophysics, Geosystems 2006, 7.

[11] Tivey MK (1998). How to build a black smoker chimney. Oceanus.

[12] Olins HC, Rogers DR, Frank KL, Vidoudez C, Girguis PR (2013). Assessing the influence of physical, geochemical and biological factors on anaerobic microbial primary productivity within hydrothermal vent chimneys. Geobiology.

[13] Sievert S, Vetriani C (2012). Chemoautotrophy at deep-sea vents: past, present, and future. Oceanography.

[14] Waite DW, Vanwonterghem I, Rinke C, Parks DH, Zhang Y, Takai K, Sievert SM, Simon J, Campbell BJ, Hanson TE (2017). Comparative genomic analysis of the class Epsilonproteobacteria and proposed reclassification to Epsilonbacteraeota (phyl. nov.). Frontiers in Microbiology.

[15] Meier DV, Pjevac P, Bach W, Hourdez S, Girguis PR, Vidoudez C, Amann R, Meyerdierks A (2017). Niche partitioning of diverse sulfur-oxidizing bacteria at hydrothermal vents. ISME J.

[16] Anderson RE, Sogin ML, Baross JA (2015). Biogeography and ecology of the rare and abundant microbial lineages in deep-sea hydrothermal vents. FEMS Microbiol Ecol.

[17] Flores GE, Campbell JH, Kirshtein JD, Meneghin J, Podar M, Steinberg JI, Seewald JS, Tivey MK, Voytek MA, Yang ZK, Reysenbach AL (2011). Microbial community structure of hydrothermal deposits from geochemically different vent fields along the Mid-Atlantic Ridge. Environ Microbiol.

[18] Zhou H, Li J, Peng X, Meng J, Wang F, Ai Y (2009). Microbial diversity of a sulfide black smoker in main endeavour hydrothermal vent field, Juan de Fuca Ridge. J Microbiol.

[19] Han Y, Gonnella G, Adam N, Schippers A, Burkhardt L, Kurtz S, Schwarz-Schampera U, Franke H, Perner M (2018). Hydrothermal chimneys host habitat-specific microbial communities: analogues for studying the possible impact of mining seafloor massive sulfide deposits. Sci Rep.

[20] Dahle H, Roalkvam I, Thorseth IH, Pedersen RB, Steen IH (2013). The versatile in situ gene expression of an Epsilonproteobacteria-dominated biofilm from a hydrothermal chimney. Environ Microbiol Rep.

[21] Stokke R, Dahle H, Roalkvam I, Wissuwa J, Daae FL, Tooming-Klunderud A, Thorseth IH, Pedersen RB, Steen IH (2015). Functional interactions among filamentous Epsilonproteobacteria and Bacteroidetes in a deep-sea hydrothermal vent biofilm. Environ Microbiol.

[22] Perner M, Gonnella G, Kurtz S, LaRoche J (2014). Handling temperature bursts reaching 464 ° C: different microbial strategies in the Sisters Peak hydrothermal chimney. Appl Environ Microbiol.

[23] Dahle H, Okland I, Thorseth IH, Pederesen RB, Steen IH (2015). Energy landscapes shape microbial communities in hydrothermal systems on the Arctic Mid-Ocean Ridge. ISME J.

[24] Fortunato CS, Huber JA (2016). Coupled RNA-SIP and metatranscriptomics of active chemolithoautotrophic communities at a deep-sea hydrothermal vent. ISME J.

[25] Takai K, Nunoura T, Horikoshi K, Shibuya T, Nakamura K, Suzuki Y, Stott M, Massoth GJ (2009). Christenson BW, deRonde CEJ, et al: Variability in microbial communities in black smoker chimneys at the NW caldera vent field, Brothers volcano, Kermadec arc. Geomicrobiol J.

[26] Delaney JR, Kelley DS, Mathez EA, Yoerger DR, Baross J, Schrenk MO, Tivey MK, Kaye J, Robigou V (2001). “Edifice Rex” sulfide recovery project: analysis of submarine hydrothermal, microbial habitat. Eos, Transactions American Geophysical Union.

[27] Steen IH, Dahle H, Stokke R, Roalkvam I, Daae FL, Rapp HT, Pedersen RB, Thorseth IH (2015). Novel barite chimneys at the Loki’s castle vent field shed light on key factors shaping microbial communities and functions in hydrothermal systems. Front Microbiol.

[28] Nakagawa S, Takaki Y, Shimamura S, Reysenbach AL, Takai K, Horikoshi K (2007). Deep-sea vent epsilon-proteobacterial genomes provide insights into emergence of pathogens. Proc Natl Acad Sci U S A.

[29] Campbell BJ, Engel AS, Porter ML, Takai K (2006). The versatile epsilon-proteobacteria: key players in sulphidic habitats. Nat Rev Microbiol.

[30] Böhnke S, Sass K, Gonnella G, Diehl A, Kleint C, Bach W, Zitoun R, Koschinsky A, Indenbirken D, Sander SG (2019). Parameters governing the community structure and element turnover in Kermadec volcanic ash and hydrothermal fluids as monitored by inorganic electron donor consumption, autotrophic CO_2_ fixation and 16S tags of the transcriptome in incubation experiments. Front Microbiol.

[31] McNichol J, Stryhanyuk H, Sylva SP, Thomas F, Musat N, Seewald JS, Sievert SM (2018). Primary productivity below the seafloor at deep-sea hot springs. Proceedings of the National Academy of Sciences.

[32] McNichol J, Sylva SP, Thomas F, Taylor CD, Sievert SM, Seewald JS (2016). Assessing microbial processes in deep-sea hydrothermal systems by incubation at in situ temperature and pressure. Deep Sea Research Part I: Oceanographic Research Papers.

[33] Li J, Cui J, Yang Q, Cui G, Wei B, Wu Z, Wang Y, Zhou H (2017). Oxidative weathering and microbial diversity of an inactive seafloor hydrothermal sulfide chimney. Front Microbiol.

[34] Kato S, Takano Y, Kakegawa T, Oba H, Inoue K, Kobayashi C, Utsumi M, Marumo K, Kobayashi K, Ito Y (2010). Biogeography and biodiversity in sulfide structures of active and inactive vents at deep-sea hydrothermal fields of the Southern Mariana Trough. Appl Environ Microbiol.

[35] Sylvan JB, Toner BM, Edwards KJ (2012). Life and death of deep-sea vents: bacterial diversity and ecosystem succession on inactive hydrothermal sulfides. MBio.

[36] Suzuki Y, Inagaki F, Takai K, Nealson KH, Horikoshi K (2004). Microbial diversity in inactive chimney structures from deep-sea hydrothermal systems. Microb Ecol.

[37] Meier DV, Pjevac P, Bach W, Markert S, Schweder T, Jamieson J, Petersen S, Amann R, Meyerdierks A (2019). Microbial metal-sulfide oxidation in inactive hydrothermal vent chimneys suggested by metagenomic and metaproteomic analyses. Environ Microbiol.

[38] Seewald JS, Doherty KW, Hammar TR, Liberatore SP (2002). A new gas-tight isobaric sampler for hydrothermal fluids. Deep Sea Research Part I: Oceanographic Research Papers.

[39] Zhang X, Morono Y, Inagaki F, Wang F (2016). A modified SDS-based DNA extraction method for high quality environmental DNA from seafloor environments. Frontiers in Microbiology.

[40] Peng Y, Leung HC, Yiu S-M, Chin FY (2012). IDBA-UD: a de novo assembler for single-cell and metagenomic sequencing data with highly uneven depth. Bioinformatics.

[41] Langmead B, Salzberg SL (2012). Fast gapped-read alignment with Bowtie 2. Nature methods.

[42] Albertsen M, Hugenholtz P, Skarshewski A, Nielsen KL, Tyson GW, Nielsen PH (2013). Genome sequences of rare, uncultured bacteria obtained by differential coverage binning of multiple metagenomes. Nat Biotechnol.

[43] Miller CS, Baker BJ, Thomas BC, Singer SW, Banfield JF (2011). EMIRGE: reconstruction of full-length ribosomal genes from microbial community short read sequencing data. Genome Biol.

[44] Quast C, Pruesse E, Yilmaz P, Gerken J, Schweer T, Yarza P, Peplies J, Glockner FO (2013). The SILVA ribosomal RNA gene database project: improved data processing and web-based tools. Nucleic Acids Res.

[45] Hyatt D, Chen G-L, LoCascio PF, Land ML, Larimer FW, Hauser LJ. Prodigal: prokaryotic gene recognition and translation initiation site identification. BMC bioinformatics 2010;11:119. 10.1186/1471-2105-11-119 PMC2848648 20211023

[46] Kanehisa M, Sato Y, Furumichi M, Morishima K, Tanabe M (2019). New approach for understanding genome variations in KEGG. Nucleic Acids Res.

[47] Haft DH, Loftus BJ, Richardson DL, Yang F, Eisen JA, Paulsen IT, White O (2001). TIGRFAMs: a protein family resource for the functional identification of proteins. Nucleic Acids Res.

[48] El-Gebali S, Mistry J, Bateman A, Eddy SR, Luciani A, Potter SC, Qureshi M, Richardson LJ, Salazar GA, Smart A (2019). The Pfam protein families database in 2019. Nucleic Acids Res.

[49] Huerta-Cepas J, Forslund K, Coelho LP, Szklarczyk D, Jensen LJ, von Mering C, Bork P (2017). Fast genome-wide functional annotation through orthology assignment by eggNOG-mapper. Mol Biol Evol.

[50] Finn RD, Clements J, Eddy SR (2011). HMMER web server: interactive sequence similarity searching. Nucleic Acids Res.

[51] Liao Y, Smyth GK, Shi W: featureCounts: an efficient general purpose program for assigning sequence reads to genomic features. Bioinformatics 2014;30:923-30. 10.1093/bioinformatics/btt656 24227677

[52] Parks DH, Beiko RG (2010). Identifying biologically relevant differences between metagenomic communities. Bioinformatics.

[53] Anantharaman K, Hausmann B, Jungbluth SP, Kantor RS, Lavy A, Warren LA, Rappe MS, Pester M, Loy A, Thomas BC, Banfield JF (2018). Expanded diversity of microbial groups that shape the dissimilatory sulfur cycle. ISME J.

[54] Muller AL, Kjeldsen KU, Rattei T, Pester M, Loy A (2015). Phylogenetic and environmental diversity of DsrAB-type dissimilatory (bi)sulfite reductases. ISME J.

[55] Katoh K, Standley DM (2013). MAFFT multiple sequence alignment software version 7: improvements in performance and usability. Mol Biol Evol.

[56] Capella-Gutiérrez S, Silla-Martínez JM, Gabaldón T (2009). trimAl: a tool for automated alignment trimming in large-scale phylogenetic analyses. Bioinformatics.

[57] Nguyen L-T, Schmidt HA, Von Haeseler A, Minh BQ (2015). IQ-TREE: a fast and effective stochastic algorithm for estimating maximum-likelihood phylogenies. Mol Biol Evol.

[58] Wang Y, Wegener G, Hou J, Wang F, Xiao X (2019). Expanding anaerobic alkane metabolism in the domain of Archaea. Nat Microbiol.

[59] Wu Y-W, Simmons BA, Singer SW (2016). MaxBin 2.0: an automated binning algorithm to recover genomes from multiple metagenomic datasets. Bioinformatics.

[60] Kang DD, Froula J, Egan R, Wang Z (2015). MetaBAT, an efficient tool for accurately reconstructing single genomes from complex microbial communities. PeerJ.

[61] Parks DH, Imelfort M, Skennerton CT, Hugenholtz P, Tyson GW (2015). CheckM: assessing the quality of microbial genomes recovered from isolates, single cells, and metagenomes. Genome Res.

[62] Parks DH, Rinke C, Chuvochina M, Chaumeil PA, Woodcroft BJ, Evans PN, Hugenholtz P, Tyson GW (2017). Recovery of nearly 8,000 metagenome-assembled genomes substantially expands the tree of life. Nat Microbiol.

[63] Dombrowski N, Teske AP, Baker BJ (2018). Expansive microbial metabolic versatility and biodiversity in dynamic Guaymas Basin hydrothermal sediments. Nat Commun.

[64] Stamatakis A (2014). RAxML version 8: a tool for phylogenetic analysis and post-analysis of large phylogenies. Bioinformatics.

[65] Letunic I, Bork P (2016). Interactive tree of life (iTOL) v3: an online tool for the display and annotation of phylogenetic and other trees. Nucleic Acids Res.

[66] Castelle CJ, Hug LA, Wrighton KC, Thomas BC, Williams KH, Wu D, Tringe SG, Singer SW, Eisen JA, Banfield JF (2013). Extraordinary phylogenetic diversity and metabolic versatility in aquifer sediment. Nat Commun.

[67] Brown CT, Olm MR, Thomas BC, Banfield JF (2016). Measurement of bacterial replication rates in microbial communities. Nat Biotechnol.

[68] Sondergaard D, Pedersen CN, Greening C (2016). HydDB: a web tool for hydrogenase classification and analysis. Sci Rep.

[69] King GM, Weber CF (2007). Distribution, diversity and ecology of aerobic CO-oxidizing bacteria. Nat Rev Microbiol.

[70] Von Damm KL, Edmond JM, Grant B, Measures CI, Walden B, Weiss RF (1985). Chemistry of submarine hydrothermal solutions at 21 °N, East Pacific Rise. Geochimica et Cosmochimica Acta.

[71] Kato S, Shibuya T, Takaki Y, Hirai M, Nunoura T, Suzuki K (2018). Genome-enabled metabolic reconstruction of dominant chemosynthetic colonizers in deep-sea massive sulfide deposits. Environ Microbiol.

[72] Mardanov AV, Beletsky AV, Kadnikov VV, Slobodkin AI, Ravin NV (2016). Genome analysis of Thermosulfurimonas dismutans, the first thermophilic sulfur-disproportionating bacterium of the phylum Thermodesulfobacteria. Front Microbiol.

[73] Kato S, Ohkuma M, Powell DH, Krepski ST, Oshima K, Hattori M, Shapiro N, Woyke T, Chan CS (2015). Comparative genomic insights into ecophysiology of neutrophilic, microaerophilic iron oxidizing bacteria. Front Microbiol.

[74] Nercessian O, Reysenbach AL, Prieur D, Jeanthon C (2010). Archaeal diversity associated with in situ samplers deployed on hydrothermal vents on the East Pacific Rise (13 degrees N). Environ Microbiol.

[75] Karine A, Magali Z, Nadine LB, Françoise L, Joël Q, Françoise G, Marie-Anne CB (2010). Early steps in microbial colonization processes at deep-sea hydrothermal vents. Environ Microbiol.

[76] Wang F, Zhou H, Meng J, Peng X, Jiang L, Sun P, Zhang C, Van Nostrand JD, Deng Y, He Z (2009). GeoChip-based analysis of metabolic diversity of microbial communities at the Juan de Fuca Ridge hydrothermal vent. Proc Natl Acad Sci U S A.

[77] Page A, Tivey MK, Stakes DS, Reysenbach AL (2008). Temporal and spatial archaeal colonization of hydrothermal vent deposits. Environ Microbiol.

[78] McCliment EA, Voglesonger KM, O'Day PA, Dunn EE, Holloway JR, Cary SC (2006). Colonization of nascent, deep-sea hydrothermal vents by a novel Archaeal and Nanoarchaeal assemblage. Environ Microbiol.

[79] Reysenbach AL, Longnecker K, Kirshtein J (2000). Novel bacterial and archaeal lineages from an in situ growth chamber deployed at a Mid-Atlantic Ridge hydrothermal vent. Applenvironmicrobiol.

[80] Wirth R (2017). Colonization of black smokers by hyperthermophilic microorganisms. Trends Microbiol.

[81] Bellack A, Huber H, Rachel R, Wanner G, Wirth R (2011). Methanocaldococcus villosus sp. nov., a heavily flagellated archaeon that adheres to surfaces and forms cell-cell contacts. Int J Syst Evol Microbiol.

[82] Huber H, Burggraf S, Mayer T, Wyschkony I, Rachel R, Stetter KO: Ignicoccus gen. nov., a novel genus of hyperthermophilic, chemolithoautotrophic Archaea, represented by two new species, Ignicoccus islandicus sp nov and Ignicoccus pacificus sp nov. and Ignicoccus pacificus sp. nov. International Journal of Systematic and Evolutionary Microbiology 2000, 50:2093-2100. 10.1099/00207713-50-6-2093 11155984

[83] Li J, Zhou H, Fang J, Sun Y, Dasgupta S (2014). Microbial distribution in different spatial positions within the walls of a black sulfide hydrothermal chimney. Mar Ecol Prog Ser.

[84] Schrenk MO, Kelley DS, Delaney JR, Baross JA (2003). Incidence and diversity of microorganisms within the walls of an active deep-sea sulfide chimney. Appl Environ Microbiol.

[85] He Y, Feng X, Fang J, Zhang Y, Xiao X (2015). Metagenome and metatranscriptome revealed a highly active and intensive sulfur cycle in an oil-immersed hydrothermal chimney in Guaymas Basin. Front Microbiol.

[86] Pjevac P, Meier DV, Markert S, Hentschker C, Schweder T, Becher D, Gruber-Vodicka HR, Richter M, Bach W, Amann R, Meyerdierks A (2018). Metaproteogenomic profiling of microbial communities colonizing actively venting hydrothermal chimneys. Front Microbiol.

[87] Barco RA, Emerson D, Sylvan JB, Orcutt BN, Jacobson Meyers ME, Ramirez GA, Zhong JD, Edwards KJ (2015). New insight into microbial iron oxidation as revealed by the proteomic profile of an obligate iron-oxidizing chemolithoautotroph. Appl Environ Microbiol.

[88] McAllister SM, Polson SW, Butterfield DA, Glazer BT, Sylvan JB, Chan CS: Validating the Cyc2 neutrophilic iron oxidation pathway using meta-omics of Zetaproteobacteria iron mats at marine hydrothermal vents. mSystems 2020, 5:e00553-00519. 10.1128/mSystems.00553-19 PMC7029218 32071158

[89] Eddie BJ, Wang Z, Hervey WJ, Leary DH, Malanoski AP, Tender LM, Lin B, Strycharz-Glaven SM: Metatranscriptomics supports the mechanism for biocathode electroautotrophy by “Candidatus Tenderia electrophaga”. mSystems 2017, 2:e00002-00017. 10.1128/mSystems.00002-17 PMC5371394 28382330

[90] Barco RA, Hoffman CL, Ramirez GA, Toner BM, Edwards KJ, Sylvan JB (2017). In-situ incubation of iron-sulfur mineral reveals a diverse chemolithoautotrophic community and a new biogeochemical role for Thiomicrospira. Environ Microbiol.

[91] Percak-Dennett E, He S, Converse B, Konishi H, Xu H, Corcoran A, Noguera D, Chan C, Bhattacharyya A, Borch T (2017). Microbial acceleration of aerobic pyrite oxidation at circumneutral pH. Geobiology.

[92] Jaeschke A, Jorgensen SL, Bernasconi SM, Pedersen RB, Thorseth IH, Fruh-Green GL (2012). Microbial diversity of Loki’s Castle black smokers at the Arctic Mid-Ocean Ridge. Geobiology.

[93] Stott MB, Saito JA, Crowe MA, Dunfield PF, Hou S, Nakasone E, Daughney CJ, Smirnova AV, Takai K, Alam M (2008). Culture-independent characterization of a novel microbial community at a hydrothermal vent at Brothers volcano, Kermadec arc, New Zealand. J Geophys Res Solid Earth.

[94] Cao H, Wang Y, Lee OO, Zeng X, Shao Z, Qian PY (2014). Microbial sulfur cycle in two hydrothermal chimneys on the Southwest Indian Ridge. MBio.

[95] Stolz JF, Basu P (2002). Evolution of nitrate reductase: molecular and structural variations on a common function. ChemBioChem.

[96] Xie W, Wang F, Guo L, Chen Z, Sievert SM, Meng J, Huang G, Li Y, Yan Q, Wu S (2010). Comparative metagenomics of microbial communities inhabiting deep-sea hydrothermal vent chimneys with contrasting chemistries. The ISME Journal.

