# Supplementary material for: Microbial succession during the transition from active to inactive stages of deep-sea hydrothermal vent sulfide chimneys
Source: Microbiome. 2020 Jun 30;8:102. doi: 10.1186/s40168-020-00851-8 (PMC7329443; doi:10.1186/s40168-020-00851-8)
Supplement: Supplementary file 2 — Additional file 1: Figure S1. Taxonomic classification of key functional genes retrieved from the L- and M-vent chimney. (a) The key genes enriched in the active L-vent chimney. (b) The key genes enriched in the recently inactive M-vent chimney. Figure S2 Maximum-likelihood phylogeny of dsrA genes retrieved from L-vent and M-vent chimney. The red branches represent the dsrA genes recovered from active the L-vent chimney, while the blue ones are those from recently inactive M-vent chimney. Numbers of dsrA gene for each sample are displayed in the parenthesis after the clade name. Figure S3 Maximum-likelihood phylogeny of soxB genes retrieved from L-vent and M-vent chimney. The red nodes represent the soxB genes recovered from the active L-vent chimney, while the blue ones are those from the recently inactive M-vent chimney. Four soxB genes from M-vent chimney form a distinct clade (marked with red star) that different with other soxB. Figure S4 Distribution and quantity of top 50 RpS3 genes retrieved in the MAGs for two chimney samples. (a) in the active L-vent chimney. (b) in the recently inactive M-vent chimney. The x-axis represents each RpS3 gene identified in two samples, the y-axis is the sequencing depth for each RpS3 gene. The red bars indicate the RpS3 genes identified in the MAGs in corresponding samples, while the blue ones are those are not included in the MAGs. Figure S5 Maximum-likelihood phylogeny of coxL genes retrieved from L- and M-vent chimney. The red branches represent the coxL genes recovered from active the L-vent chimney, while the blue ones are those from recently inactive M-vent chimney. Support for internal nodes was constructed from 1000 bootstrap replicates, black dots represent those nodes with bootstrap value > 75%, which is direct proportional to their diameter. Figure S6 Phylogeny of 6 Nitrospirae MAGs recovered from M-vent chimney. The phylogenetic tree is based on 37 concatenated ribosomal proteins and collapsed at the genus level. [file 40168_2020_851_MOESM1_ESM.pdf]

Additional file 1

Microbial succession during the transition from active to inactive stages of deep-sea hydrothermal vent sulfide chimneys

Jialin Hou, Stefan M. Sievert, Yinzhao Wang, Jeff S. Seewald, Vengadesh Perumal Natarajan

Fengping Wang*, Xiang Xiao*

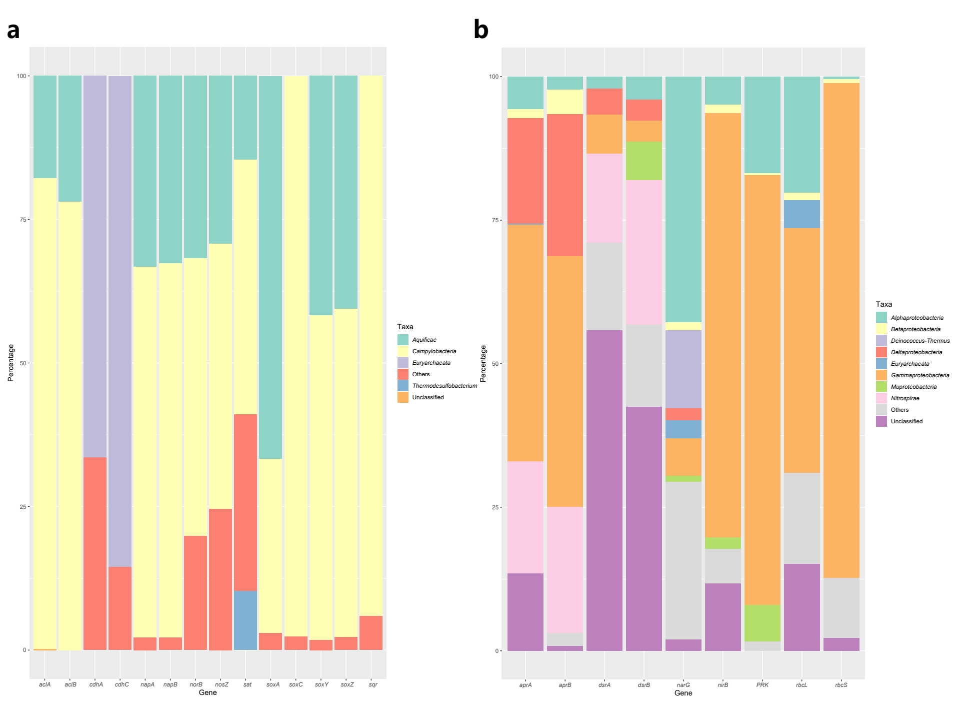

Figure S1 Taxonomic classification of key functional genes retrieved from the L- and M-vent chimeny.(a) The key genes enriched in the active L-vent chimney. (b) The key genes enriched in the recently inactive M-vent chimney.

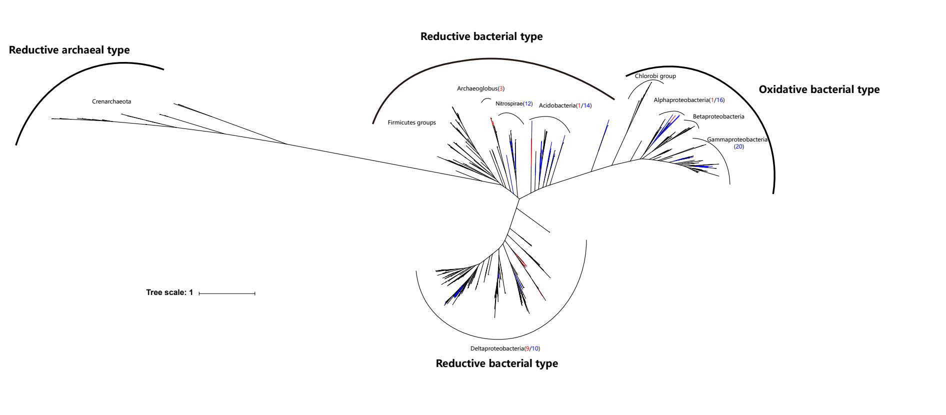

Figure S2 Maximum-likelihood phylogeny of *dsrA* genes retrieved from L- and M-vent chimney. The red branches represent the *dsrA* genes recovered from active the L-vent chimney, while the blue ones are those from recently inactive M-vent chimney. Numbers of *dsrA* gene for each sample are displayed in the parenthesis after the clade name.

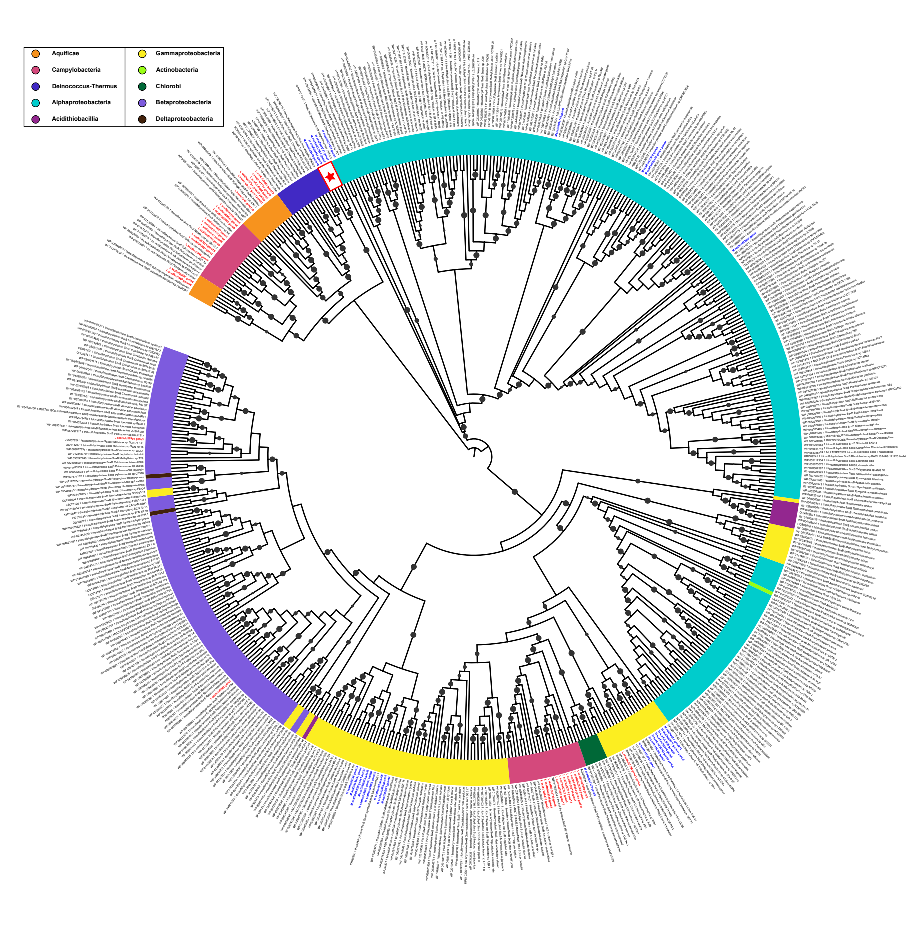

Figure S3 Maximum-likelihood phylogeny of *soxB* genes retrieved from L- and M-vent chimney. The red nodes represent the *soxB* genes recovered from the active L-vent chimney, while the blue ones are those from the recently inactive M-vent chimney. Four *soxB* genes from M-vent chimney form a distinct clade (marked with red star) that different with other *SoxB*.

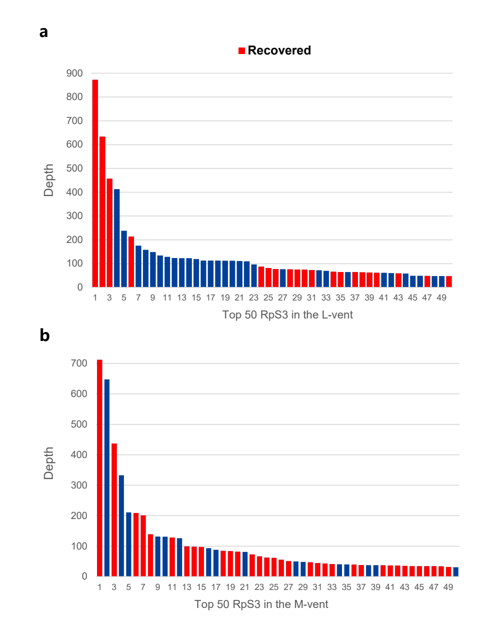

Figure S4 The distribution and quantity of top 50 *RpS3* genes retrieved in the MAGs (metagenomic assembled genomes) for two chimney samples. (a) in the active L-vent chimeny. (b) in the recently inactive M-vent chimney. The x-axis represents each *RpS3* gene identified in two samples, the y-axis is the sequencing depth for each *RpS3* gene. The red bars indicate the *RpS3* genes identified in the MAGs in corresponding samples, while the blue ones are those are not included in the MAGs.

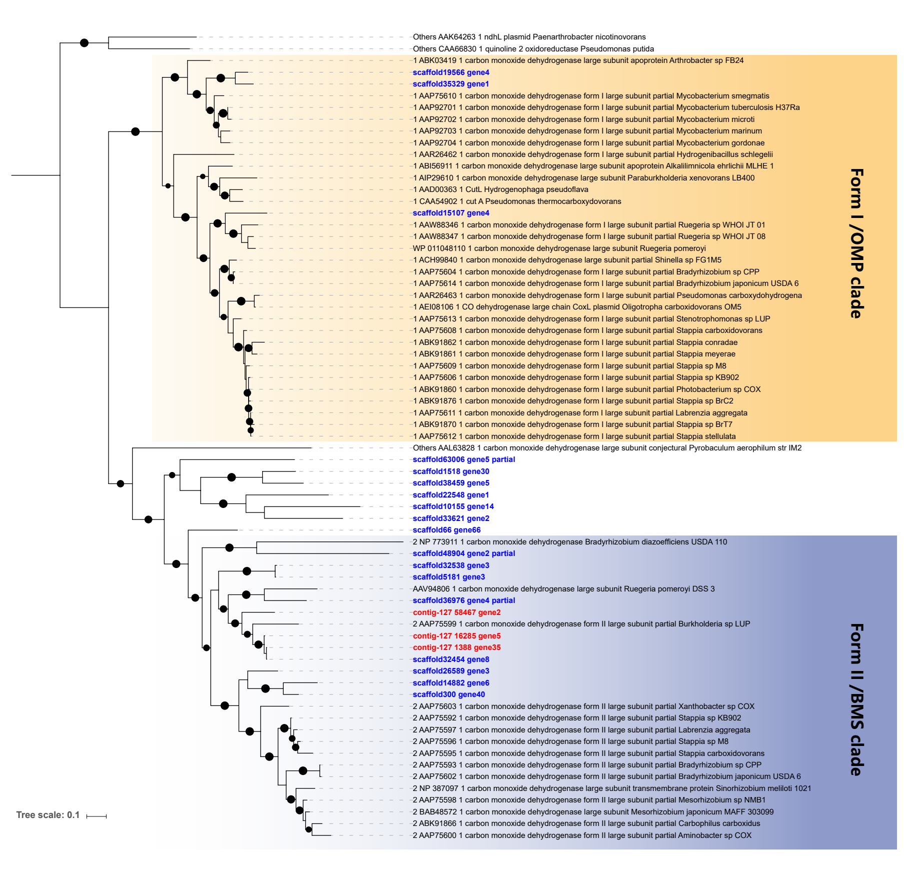

Figure S5 Maximum-likelihood phylogeny of *coxL* genes retrieved from L- and M-vent chimney. The red branches represent the *coxL* genes recovered from active the L-vent chimney, while the blue ones are those from recently inactive M-vent chimney. Support for internal nodes was constructed from 1000 bootstrap replicates, black dots represent those nodes with bootstrap value > 75%, which is direct proportional to their diameter.

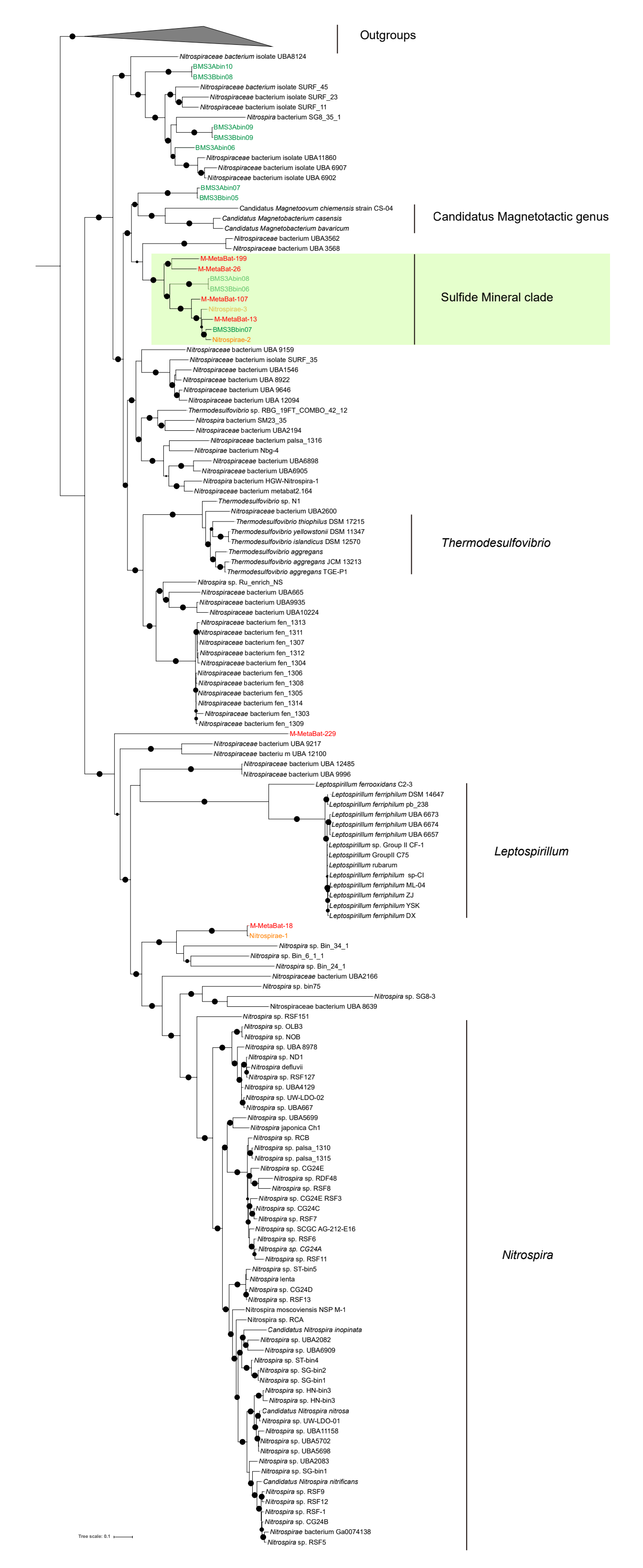

Figure S6 Phylogeny of 6 *Nitrospirae* MAGs (metagenomic assembled genomes) recovered from M-vent chimney. The phylogenetic tree is based on 37 concatenated ribosomal proteins and collapsed at the genus level. The red nodes represent those MAGs recovered from M-vent chimney, the green ones are those from subseafloor massive sulfide deposits [72] and the organe ones are those retrieved from inactive chimneys [37]. The green-shded branches are proposed as “Sulfide mineral” clade of *Nitrospirae* in this study.

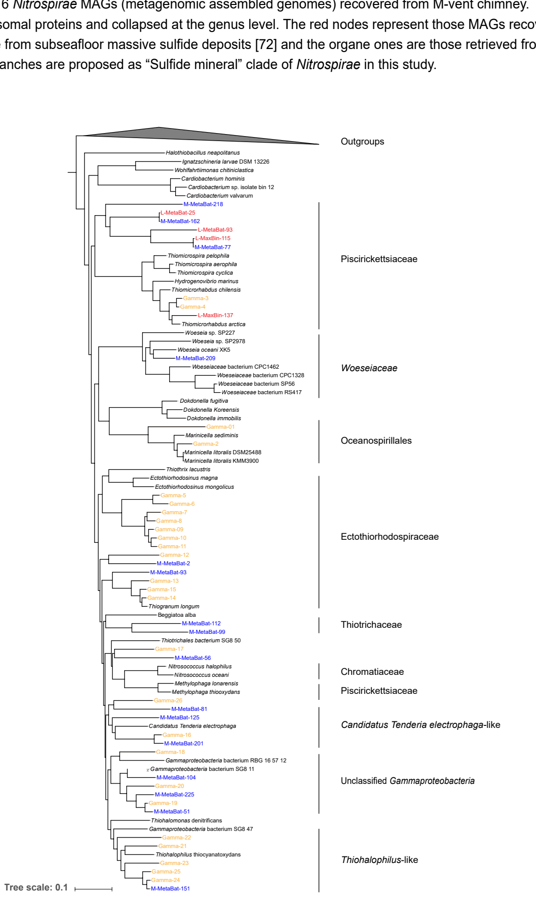

Figure S7 Phylogeny of 20 *Gammaproteobacteria* MAGs (metagenomic assembled genomes) recovered from M- and L-vent chimney. The phylogenetic tree is based on 37 concatenated ribosomal proteins and collapsed at the genus level. The red nodes represent those MAGs recovered from L-vent chimney, and blue for M-vent chimney. The organe ones are those retrieved from inactive chimneys [37].

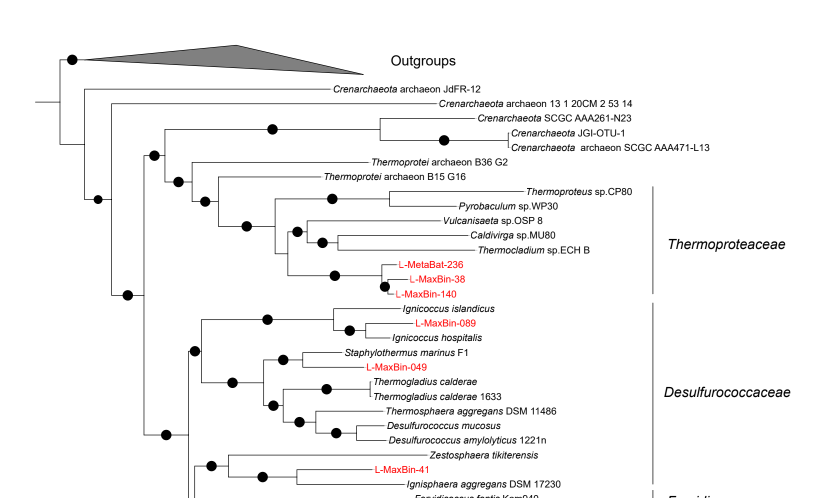

Figure S8 Phylogeny of 8 *Crenarchaeota* MAGs (metagenomic assembled genomes) recovered from L-vent chimney. The phylogenetic tree is based on 37 concatenated ribosomal proteins and collapsed at the family level. The red nodes represent those MAGs recovered from L-vent chimney.

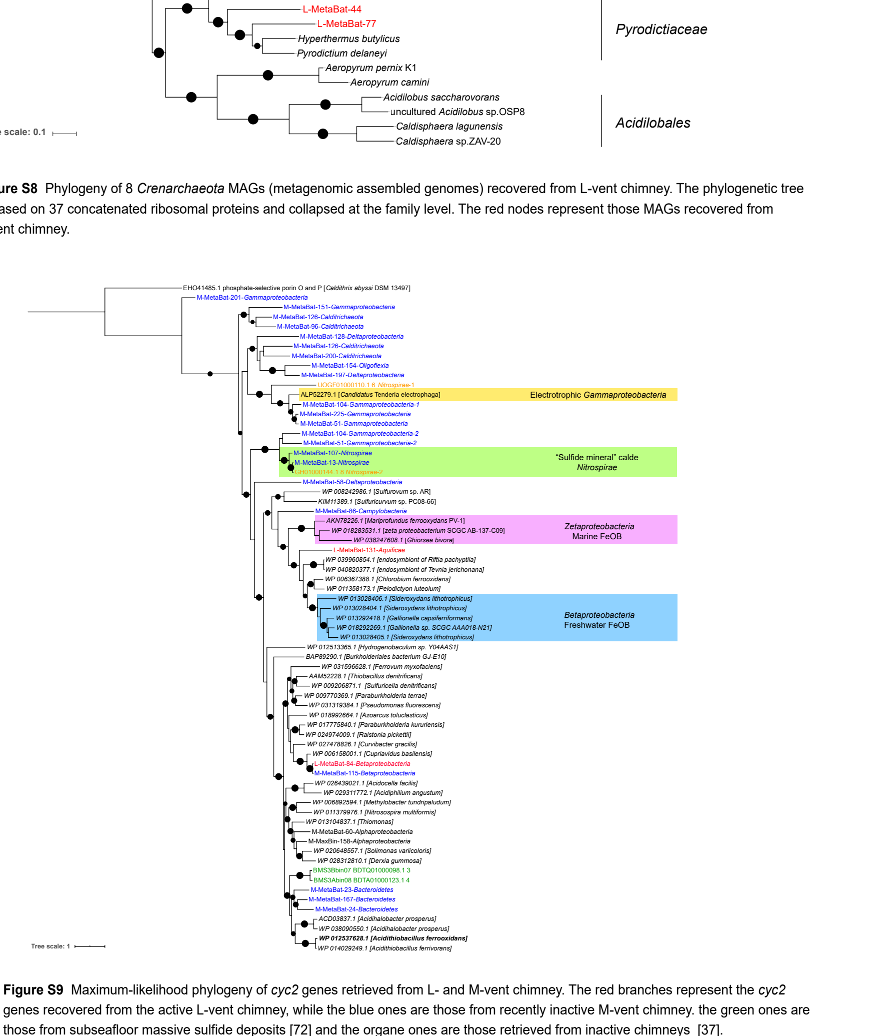

Figure S9 Maximum-likelihood phylogeny of *cyc2* genes retrieved from L- and M-vent chimney. The red branches represent the *cyc2* genes recovered from the active L-vent chimney, while the blue ones are those from recently inactive M-vent chimney. the green ones are those from subseafloor massive sulfide deposits [72] and the organe ones are those retrieved from inactive chimneys [37]. The other reference sequences are come from Kato *et al.,* 2015 [74]. Support for internal nodes was constructed from 1000 bootstrap replicates, black dots represent those nodes with bootstrap value > 70%, which is direct proportional to their diameter.
